# Patterned Arteriole-Scale Vessels Enhance Engraftment, Perfusion, and Vessel Branching Hierarchy of Engineered Human Myocardium for Heart Regeneration

**DOI:** 10.3390/cells12131698

**Published:** 2023-06-23

**Authors:** Rajeev J. Kant, Kiera D. Dwyer, Jang-Hoon Lee, Collin Polucha, Momoka Kobayashi, Stephen Pyon, Arvin H. Soepriatna, Jonghwan Lee, Kareen L. K. Coulombe

**Affiliations:** School of Engineering, Brown University Center for Biomedical Engineering, Providence, RI 02912, USA; rajeev_kant@brown.edu (R.J.K.);

**Keywords:** inosculation, vascularization, hiPSC-derived cardiomyocytes, patterned vessels, engineered cardiac tissue, heart regeneration

## Abstract

Heart regeneration after myocardial infarction (MI) using human stem cell-derived cardiomyocytes (CMs) is rapidly accelerating with large animal and human clinical trials. However, vascularization methods to support the engraftment, survival, and development of implanted CMs in the ischemic environment of the infarcted heart remain a key and timely challenge. To this end, we developed a dual remuscularization-revascularization therapy that is evaluated in a rat model of ischemia-reperfusion MI. This study details the differentiation of human induced pluripotent stem cell-derived cardiomyocytes (hiPSC-CMs) for engineering cardiac tissue containing patterned engineered vessels 400 μm in diameter. Vascularized engineered human myocardial tissues (vEHMs) are cultured in static conditions or perfused in vitro prior to implantation and evaluated after two weeks. Immunohistochemical staining indicates improved engraftment of hiPSC-CMs in in vitro-perfused vEHMs with greater expression of SMA+ vessels and evidence of inosculation. Three-dimensional vascular reconstructions reveal less tortuous and larger intra-implant vessels, as well as an improved branching hierarchy in in vitro-perfused vEHMs relative to non-perfused controls. Exploratory RNA sequencing of explanted vEHMs supports the hypothesis that co-revascularization impacts hiPSC-CM development in vivo. Our approach provides a strong foundation to enhance vEHM integration, develop hierarchical vascular perfusion, and maximize hiPSC-CM engraftment for future regenerative therapy.

## 1. Introduction

Myocardial infarction (MI) causes an interruption of vascular blood flow to downstream muscles, resulting in ischemia and necrosis of cardiac tissue. Approaches in regenerative medicine to implant human induced pluripotent stem cell-derived cardiomyocytes (hiPSC-CMs) seek to repopulate the infarct zone with functional tissue that can mechanically reinforce the scar-laden ventricle, coordinate with and augment contractile function, and ultimately prevent further functional deterioration and end-stage heart failure [1]. Many leading investigators in the field hypothesize that a robust vascular supply is needed to ensure long-term engraftment and survival of implanted cardiomyocytes, particularly in the case of epicardial patch-based approaches where highly dense tissues are initially sequestered from the host vasculature [2,3]. Therefore, novel strategies to elicit rapid perfusion of engineered cardiac tissues after implantation are critically needed.

Strategies to prevascularize cardiac tissues in vitro aim to provide conduits for rapid perfusion after implantation [4]. As opposed to in situ methods, which necessitate host vasculature to grow into and perfuse an implant [2,5], in vitro prevascularization provides the opportunity for a high degree of control over engineered cardiac tissue design, intentional vascular development, [4] and may function to improve in vivo engraftment of muscle [6]. Common methods include co-culture of vascular units (e.g., endothelial cells (ECs) [7] or isolated microvessels [8]) to establish homogeneously distributed microvasculature or patterning vessels across larger scales for top-down vascular integration by way of microfabrication techniques such as 3D bioprinting [9,10,11] and pattern templating [12,13,14]. Host-driven integration of implanted vascular designs with native vasculature in vivo is termed inosculation, or the natural connection and perfusion of disparate vascular beds [15], which is in contrast to direct surgical anastomosis of pre-formed vessels capable of suture retention and withstanding systolic pressure.

Evaluating the effectiveness of vascularization techniques has historically relied on the gold standard of static 2D histological analysis, and newer methodologies show promise in providing greater insight into vascular remodeling in the infarcted heart [4]. Techniques including micro-computed tomography (microCT)-assisted reconstructions [16,17], optical coherence tomography (OCT) imaging [12,13,18], and fluorescence imaging [19,20,21] enable vascular visualization down to the capillary scale and mapping of perfused vessels. The continued development of such techniques in the context of heart regeneration will be invaluable in evaluating the in vivo effectiveness of in vitro-perfused engineered cardiac tissues.

As the sophistication of cardiac regenerative therapies advances, several considerations must be balanced for design and implementation. First, differentiated hiPSC-CMs exhibit an immature, fetal-like phenotype [22,23,24]. While many groups have used mechanical [25,26], electrical [27], or biomolecular [28,29,30,31] methods to mature the contractile apparatus, gene expression, electrophysiology, and metabolism of hiPSC-CMs, the appropriate level of maturity for transplantation in vivo that maximizes survival (e.g., in relative ischemia) and contractile function has not been established. Recent literature suggests that immature hiPSC-CMs may engraft better onto the injured heart [32], which is thought to be due to their relatively immature metabolic profile [33], although in vitro-matured hiPSC-CMs that survive implantation appear to have more adult-like structural features [24,34]. hiPSC-CMs also mature in vivo [34,35,36], so continued examination of the relationship between cardiomyocyte maturation state at implantation and engraftment success is necessary. Second, heterocellular tissue constructs require careful consideration of culture conditions, yet uniquely optimized growth medium formulations for CMs, ECs, and other cell types imply that a single culture medium is sub-optimal for other cell types, and therefore appropriate compromises must be made. Empirical evaluation of co-culture conditions is sparse in the tissue engineering literature, yet it is of broad concern and warrants study. Finally, the throughput and ease of scalable biomanufacturing are slowed by increasingly complex tissue and vascular geometries [4,37] and challenged by the fundamental difficulties in handling hiPSC-CMs and requiring high CM density for syncytium formation [31,38,39]. These considerations have influenced the design of our vascularized engineered human myocardial tissues (vEHMs), from biomaterial selection and vessel patterns to methods of endothelialization, culture, and implantation. 

To date, no group has reported on the effects of mixed medium culture on hiPSC-CMs or the use of large vessels (400 μm diameter) in dense hiPSC-CM tissue perfused in vitro and implanted in the injured rat heart. We hypothesized that in vitro-perfused hiPSC-CM tissues with arteriole-scale, EC-lined vessels would facilitate greater perfusion of implanted hiPSC-CMs and improve their survival and maturation in vivo. Here, we report the formation of vEHMs containing patent and perfused arteriole-scale vessels. We demonstrate their robust engraftment onto infarcted myocardium with increased hiPSC-CM engraftment density and improved hierarchical vascularization of vEHMs with prior in vitro perfusion (P vEHM) compared to non-perfused (NP vEHM) or non-vascularized control tissues (EHM). Lastly, we explore mechanistic connections between engineered vasculature and implanted hiPSC-CM development through bulk RNA sequencing of explanted vEHMs.

## 2. Materials and Methods

### 2.1. hiPSC Maintenance

The WTC-11 GCaMP6f hiPSC line was used in all experiments (Dr. Bruce Conklin, The Gladstone Institutes, San Francisco, CA, USA). hiPSCs were maintained on 10 cm^2^ dishes coated with 5 μg/mL vitronectin (Thermo Fisher, Waltham, MA, USA) in a cell culture incubator (37 °C, 5% CO_2_). hiPSCs were maintained in Essential 8 (E8) medium (Gibco, Waltham, MA, USA) and passaged every 4–5 days at 80% confluency with versene (0.5 M EDTA (Fisher, Waltham, MA, USA) and 1.1 mM D-glucose (MilliporeSigma, Cleveland, OH, USA) in DPBS without calcium and magnesium (Gibco)) onto new vitronectin-coated plates.

### 2.2. hiPSC-CM Differentiation, Expansion, and Lactate Selection

**Figure 1:**
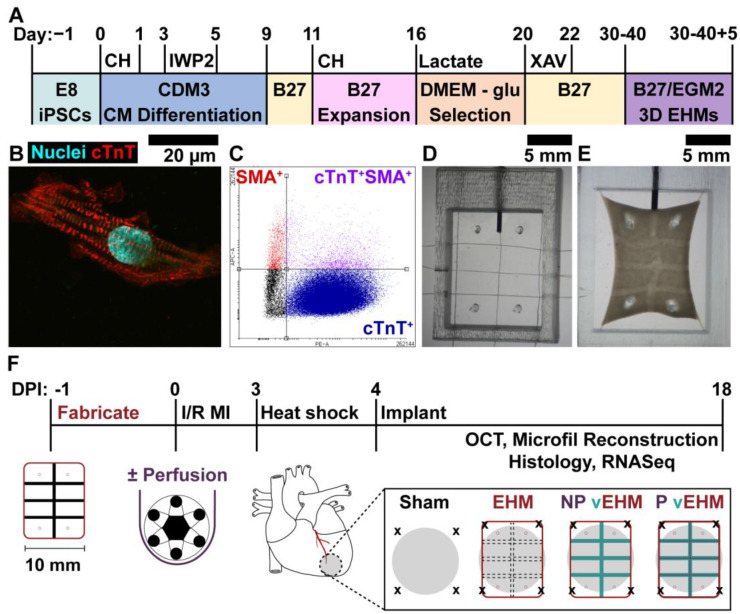
Overview of hiPSC-CM differentiation, vEHM formation, and in vivo implantation. (**A**) Differentiation timeline of hiPSCs into (**B**) ventricular CMs using multi-stage biphasic Wnt modulation. (**C**) Differentiation purity is evaluated using flow cytometry staining for cTnT. (**D**,**E**) PDMS-based replica molding system for embedding biomaterials and forming vEHMs. (**F**) Overview of timeline for in vivo implantation of EHMs in a rat model of ischemia-reperfusion MI. CH: Chiron; IWP2: Inhibitor of Wnt Production 2; XAV: XAV939; E8: Essential 8 medium; CDM3: Cardiac differentiation medium 3; glu: Glucose; B27: RPMI 1640 + B27 supplement; EGM-2: Endothelial growth media-2. cTnT: Cardiac troponin T; SMA; Smooth muscle actin; I/R MI: Ischemia-reperfusion myocardial infarction; OCT: Optical Coherence Tomography; RNASeq: Bulk RNA sequencing.

Directed differentiation of hiPSCs into cardiomyocytes (Figure 1A) was achieved via multi-stage biphasic modulation of the Wnt signaling pathway in defined conditions [40,41,42,43]. Briefly, hiPSCs were replated on Matrigel^®^-coated (Corning, Corning, NY, USA) 24 well plates in E8 with 10 μM Y-27632 (Rock Inhibitor, RI; Tocris) at a density of 40,000–70,000 cells/cm^2^. The next day (D0), hiPSCs were treated with 4–5 μM CHIR 99021 (Chiron; Tocris, Bristol, UK) in CDM3 medium [41] for 24 h. On day 3 (72 h after Chiron treatment), cells were treated with a 5 μM inhibitor of WNT production 2 (IWP2; Tocris) for 2 days, then fed CDM3 every other day until beating commenced (days 8–12).

On day 9, hiPSC-CMs were fed RPMI 1640 with B27 supplement (RPMI B27; Gibco) for two days, then on day 11, harvested with TrypLE Select 10x (Gibco) and re-plated on Matrigel^®^-coated 15 cm^2^ plates at 25,000 cells/cm^2^ in RPMI B27 containing 2 μM Chiron and 10 μM RI to encourage cardiomyocyte expansion [43]. hiPSC-CMs were fed fresh RPMI B27 with 2 μM Chiron every 2 days until 90% confluent (4–6 days). Cells were then washed with DPBS and fed DMEM—glucose (Gibco) containing 4 mM sodium lactate (MilliporeSigma) every other day for 4 days to eliminate non-cardiomyocytes from culture [28], followed by two days of treatment with 4 μM XAV939 (Tocris) in RPMI B27 to encourage cell cycle exit of hiPSC-CMs [34]. Expanded, purified hiPSC-CMs (Figure 1B) were then maintained in RPMI B27 containing 100 μg/mL penicillin-streptomycin (pen-strep; MilliporeSigma) and used between days 30–40 of differentiation for all downstream experiments (Supplementary Video S1). The average purity of hiPSC-CMs used for in vitro and in vivo experiments was 94.0% and 85.05%, respectively, by flow cytometry staining for cardiac troponin T (cTnT; Figure 1C).

### 2.3. Cardiac Fibroblast Mainenance

Primary normal adult ventricular human cardiac fibroblasts (hCFs; MilliporeSigma) were maintained on 15 cm^2^ culture dishes in hCF medium consisting of DMEM\F-12, 10% fetal bovine serum (FBS; Gibco), 4 ng/mL bFGF (Stemgent, Beltsville, MD, USA), and 100 μg/mL pen-strep. hCFs were passaged every 4–5 days at 80% confluency using 0.05% trypsin (Gibco) in versene and frozen back in hCF medium with 10% dimethyl sulfoxide (DMSO; Fisher). All experiments used hCFs directly from the thaw between passages 3–6.

### 2.4. Endothelial Cell Mainenance

Human umbilical vein endothelial cells (ECs; Lonza) were maintained on 0.1% gelatin-coated (MilliporeSigma) 15 cm^2^ culture dishes in EGM-2 Medium (Lonza, Basel, Switzerland). ECs were passaged every 4–5 days at 80% confluency using 0.05% trypsin in versene and frozen back in EGM-2 with 10% DMSO. All experiments used ECs between passages 3–7.

### 2.5. Cellular Viability, Proliferation, and Morphological Assays

EC and hiPSC-CM viability was assessed by plating cells on 0.1% gelatin-coated or Matrigel^®^-coated 6 well plates, respectively, and feeding with either control medium (EGM-2 for ECs; 0/100, or RPMI B27 for hiPSC-CMs; 100/0), or mixed medium consisting of either 70% RPMI B27 and 30% EGM-2 (70/30) or 50% RPMI B27 and 50% EGM-2 (50/50). An amount of 100 μg/mL pen-strep was added to all media. Daily images were taken to assess the number of live and dead cells in five separate microscope fields per condition. The morphology and structure of hiPSC-CMs were assessed with immunohistochemical staining.

### 2.6. Mechanical Characterization of EHMs

Culture molds were fabricated using a replica molding technique [44,45]. Briefly, vector-based designs were created in Adobe Illustrator (Adobe Inc., San Jose, CA, USA) with a 9 mm × 3 mm culture area and laser-cut into a 1/4-inch acrylic sheet using a Universal Laser 8 Systems 6.75 Laser Cutter (ULS) at the Brown Design Workshop (Brown University). Sylgard 184 (Dow) was cast into the acrylic templates and cured overnight at 60 °C. The cured PDMS molds were then removed from the acrylic negative and sterilized in an autoclave at 121 °C for 30 min. hiPSC-CMs were harvested between 30 and 40 days of differentiation and resuspended at a density of 1.5 × 10^7^ cells/mL and mixed with 5% hCFs from thawing by number and rat tail-derived collagen-1 (final concentration 1 mg/mL; Advanced BioMatrix). The suspension was cast into each trough of the PDMS mold and allowed to set for 30 min at 37 °C before feeding with either 100/0, 70/30, or 50/50 medium. Tissues were cultured for 1 week and paced at 1 Hz using a C-Pace EM Stimulator (IonOptix, Westwood, MA). Mechanical testing was performed as previously described [46]. Briefly, EHMs were mounted between a 5 mN load cell (Aurora Scientific, Aurora, Canada) and a high-speed length controller in a temperature-controlled bath of Tyrode’s solution. Initial length (L_0_) was set at just above slack length. Tissues were stretched in 5% length step increments to 130% of L_0,_ and the resulting force generation at each step was measured under a 1 Hz field stimulus. Tissues were then electrically paced from 1 Hz to 4 Hz in 0.5 Hz increments to determine the maximum capture rate before returning length to L_0_. The tissues were then immediately fixed for downstream immunohistochemistry. Passive stiffness and active force kinetics were measured using a custom MATLAB code (Mathworks Inc., Natick, MA, USA).

### 2.7. Gelatin-Alginate Fiber Wet Spinning

Wet spinning of sacrificial fibers was adapted from a previous publication [47]. Briefly, 1% *w*/*v* sodium alginate (MilliporeSigma) was prepared in DI water and loaded into a 5 mL syringe fitted with a 30G hypodermic needle. A crosslinking solution was made by dissolving 100 mM calcium chloride (Acros Organics, Waltham, MA, USA) and 2.5% *w*/*v* gelatin (MilliporeSigma) in DI water. Alginate was extruded into the crosslinking bath via a syringe pump at a rate of 50 μL/sec, and the resulting cross-linked gelatin-alginate (GA) alginate fibers were collected on a mandrel. Wet spun fibers were sprayed briefly with 70% ethanol to disinfect and stored in a biosafety cabinet. GA fiber diameter was measured using ImageJ. Node measurements were determined by counting the number of nodes that appeared normalized over the length of the coated fiber.

### 2.8. Collagen Microfiber Wet Spinning

Collagen microfiber wet spinning was adapted as previously described [48] to create bundled collagen microfibers. Briefly, 13 mg/mL of collagen-1 isolated from rat tails was extruded using a syringe pump through a spinneret and ensheathed in a coaxial flow of a high-viscosity polyethylene glycol-based neutralization buffer to form collagen microfibers. The formed microfibers were then washed in a bath of 70% ethanol and collected on a mandrel to dry. Microfiber bundles were formed by wrapping collagen microfibers around the same location on the mandrel four times and briefly rewetting the fibers with 70% ethanol to form robust bundles. Microfiber bundle diameter was measured in ImageJ.

### 2.9. EHM Fabrication for In Vivo Studies

PDMS molds were fabricated to create 10 mm × 12 mm EHMs for implantation studies and prepared as described above, with the addition of a custom inlet needle inserted mid-height into the PDMS wall prior to autoclaving. In the biosafety cabinet, GA fibers and collagen microfibers were aseptically embedded in autoclaved PDMS molds [47] using a 25G needle to form an array pattern of GA fibers through the center of the tissue aligned with the inlet needle and a set of collagen microfibers along the longitudinal edges of the mold (Figure 1D). The molds were then adhered to the bottom of untreated 6 well plates with silicone adhesive (Dow). A casting mix of hiPSC-CMs (1.5 × 10^7^ cells/mL), 5% hCFs, and 1 mg/mL collagen-1 was prepared as described above with volumes scaled to accommodate the larger culture molds and kept on ice. Concomitantly, ECs were thawed, resuspended at a density of 2.5 × 10^7^ cells/mL in a coating solution of EGM-2, 1% *w*/*v* gelatin, 0.25% *w*/*v* alginate, and 1 mg/mL collagen-1, and carefully pipetted onto the GA fibers to coat the surface. The casting mix of hiPSC-CMs, hCFs, and collagen-1 was then immediately seeded into the PDMS mold around the fibers and allowed to gel for 30 min at 37 °C to form vEHMs (Figure 1E). vEHMs were maintained in a 50/50 medium and paced at 1 Hz under field stimulation. Control EHMs were fabricated following the same procedure, except the coating solution was devoid of ECs.

### 2.10. GA Fiber Un-Crosslinking and Perfusion Culture

After tissue formation overnight, GA fibers embedded in tissues were un-crosslinked by a 3 h incubation in un-crosslinking medium consisting of calcium-free DMEM (Gibco), 2 mM GlutaMAX (Gibco), 1.5 mM sodium citrate (Fisher), and 100 μg/mL pen-strep. Un-crosslinking medium was then manually perfused through the channels to wash out any remaining GA and confirm patency, followed by a medium change back into 50/50 medium.

Dynamic perfusion culture of patterned vessels in vEHMs was achieved with the use of a custom-built Arduino-controlled peristaltic pump, enabling independent recirculating perfusion of up to three tissues simultaneously in vitro. The medium was directed through a reservoir prior to infusion to prevent embolism by bubbles and dampen the flow profile to an average flow rate of 7.28 mL/h. Medium was partially exchanged (approximately half of the total recirculating volume) every other day by replacing medium buildup in the well of the plate. Control and non-perfused tissues were not connected to perfusion devices and were fed every other day with 50/50 medium. vEHM compaction and patterned vessel diameter were evaluated using ImageJ. Perfusion device design files are free to access and download at the Harvard Dataverse at https://doi.org/10.7910/DVN/KARO3F (accessed on 30 April 2022).

### 2.11. Implantation of EHMs on Infarcted Hearts and Explant

All animal procedures are approved under IACUC #23-02-0003 at Brown University. Nude, athymic rats (8 weeks, male, 200 g) underwent ischemia-reperfusion MI (Figure 1F) using protocols published previously [2,44]. Briefly, rats were anesthetized with 2–3% isoflurane, then induced with 100 mg/kg ketamine and 90 mg/kg xylazine and placed on a ventilator. A thoracotomy was performed to expose the heart, and the left anterior descending artery was ligated with a 4-0 polypropylene suture for 60 min to induce ischemic MI, followed by reperfusion by removal of the suture and closure of the chest.

Three days after MI, EHMs were prepared for implantation by performing a heat shock protocol [49], consisting of incubation at 42 °C for 1 h. Animals were additionally given daily subcutaneous injections of 30 mg/kg cyclosporine-A to mitigate graft rejection and mitochondrial permeability transition pore opening starting the day before implantation. The next morning (four days after MI, after acute inflammation, and at peak granulation tissue [50,51]), EHMs were treated with 100 ng/mL IGF-1 (RD Systems) and 200 nM cyclosporine-A (MilliporeSigma) one hour prior to the implant procedure. Animals underwent a second thoracotomy to implant EHMs over the infarcted region of the left ventricle. Sham animals underwent both surgeries and received sutures but no implants. Echocardiography measurements were performed under mild sedation using a Vivid 7-dimensional ultrasound system (GE) with a pediatric 10 S 4–10 MHz transducer. Longitudinal and short-axis views of the left ventricle at the mid-papillary level were taken to assess cardiac function before MI (baseline), 4 days post-injury (DPI; prior to implantation), and before sacrifice at 18 DPI (2 weeks after implantation). Echocardiography data was additionally used as exclusion criteria (fractional shortening (FS) < 45%) to ensure consistent MIs with reduced FS.

After 14 days, hearts were prepared for vascular morphological and reconstructive analysis [2,16]. Briefly, rats were injected with 750 USP/mL Heparin (Meitheal Pharmaceuticals, Chicago, IL, USA) via tail vein injection, then underwent a thoracotomy to expose the heart and aorta. The superior vena cava and innominate, left common carotid, and left subclavian branches of the aortic arch were ligated, then the abdominal aorta was cannulated with an 18G catheter to provide anterograde perfusion access to the cardiac microcirculation via the aortic root. The cannula was connected to a perfusion system, and the inferior vena cava was cut to provide an outlet. Perfusion buffer containing 1 g/L adenosine (MilliporeSigma) in DPBS was perfused for 3 min to clear blood, then briefly perfused with 150 mM potassium chloride (Fisher) to arrest the heart in diastole. After another 2 min of perfusion buffer, 4% paraformaldehyde (PF; MilliporeSigma) was perfused for 10 min to fix the vasculature, followed by explantation of the heart, lungs, and aortic arch, which were fixed overnight in 4% PF.

### 2.12. Optical Coherence Tomography Angiography

In preparation for ex vivo microangiography, hearts were re-cannulated at the aortic arch using a male luer adapter prior to the innominate branch, connected to a perfusion system, and perfused with 20% Intralipid (Fresenius Kabi, Bad Homburg, Germany) solution at a pressure of 50–60 mmHg for spectral domain optical coherence tomography imaging (OCT; Thorlabs, Newton, NJ, USA). OCT acquisition utilized a large-bandwidth near-infrared (NIR) light source with a 2048-pixel line-scan camera to achieve 147,000 A-scans/s focused on areas approximately 100–200 μm below the surface of the heart or implant. A 5× objective (3 × 3 mm FOV) or 10× objective (1.5 × 1.5 mm FOV) was used for image acquisition. For each acquisition phase, 2–4 B-scans were taken at each y position with 20 volumetric scans each. A custom contrast-mask averaging process was used to process raw imaging data and remove visual artifacts due to movement. Tortuosity measurements were performed by measuring the length of a vessel initiating and terminating at branch points and normalizing by the shortest distance between the start and end. Diameter measurements from microangiograms were quantified manually in ImageJ.

### 2.13. Vascular Reconstructive Analysis and Immunohistochemistry

After OCT microangiography, hearts were cleared of Intralipid, then manually perfused with Microfil^®^ (Flow Tech Inc., South Windsor, CT, USA), allowed to cure, and scanned with a microCT imaging system (Quantum GX2) at a voxel size of 50 μm. Raw image data was processed using custom MATLAB code and segmented in SimVascular [52] to isolate implant vasculature using histological stains to determine boundaries. Host-implant bridging was manually determined from SimVascular segmentations. Vasculature was then post-processed using a connectivity algorithm to restore broken vessels due to air bubbles during Microfil^®^ perfusion and binarization artifacts [53]. The resulting vasculature was reconstructed for visualization and quantitative analysis, including vessel segment length, diameter, branching, and volume.

After scanning, hearts were sliced into 2 mm slices, processed into paraffin or frozen blocks, and sectioned into 14 μm slices for immunohistochemical analysis. Infarct size was visualized and quantified using picrosirius red—fast green stains of heart sections at mid-papillary level. Sections were treated with primary antibodies overnight at 4 °C, followed by secondary antibodies and nuclear counterstain for 1 h at room temperature. Cover slips were mounted using ProLong AntiFade Glass Mountant (Invitrogen). Histological stains were imaged using an Olympus FV3000 Confocal Microscope or an Olympus FV200 Slide Scanner (Olympus; Leduc BioImaging Facility, Brown University) and processed in ImageJ. Primary and secondary antibodies used are provided in Supplementary Table S1. Human cell density and viable implant size were measured in ImageJ from histological stains of cTnT and human-specific Ku80 (Hu-Ku80) and normalized by the quantified area of human grafts. Engraftment was determined by normalizing density measurements to implant size. Proliferation was assessed by normalizing human-specific Ki67-positive (Hu-Ki67) nuclei to total hiPSC-CM nuclei. Myosin light chain 2 (MLC-2) measurements were determined in ImageJ by thresholding histological stains of atrial (MLC-2a) and ventricular (MLC-2v) isoforms and quantifying area. Sarcomere length was determined in histological stains of cTnT by measuring the length of between 3 and 10 sarcomeres in a straight line and dividing by the number of encompassed sarcomeres. Vessel density and diameter were quantified in histological stains of isolectin B4 and Hu-Ku80 in the remote (healthy), infarct, and implant regions of each heart and normalized by imaged area. Smooth muscle actin (SMA) quantification was performed in viable implant areas by cross-registering sections with adjacent Hu-Ku80 stains and normalizing to total vasculature.

### 2.14. Exploratory Bulk RNA Sequencing

A subset of implants were explanted at the 14-day timepoint for immediate RNA isolation using tri reagent solution (Invitrogen, Waltham, MA, USA), followed by on-column processing and washing using a Reliaprep RNA Miniprep System (Promega, Madison, WI, USA). Yield and purity were analyzed on a Nanodrop ND-1000 UV-Vis Spectrophotometer (Thermo Fisher) prior to shipment for next-generation sequencing (Azenta Life Sciences, Burlington, MA, USA). The RIN score of all samples was verified to be above 7.5 before continuing with library preparation and RNA sequencing (RNAseq).

The open-source web-based platform Galaxy [54] was used to perform standard quality control analysis, trimming, alignment of the RNAseq raw data to appropriate reference genomes, and generation of count matrices. Sequence quality was assessed through FASTQC, and trimming of the Illumina adaptor sequences was performed with Trimmomatic [55]. Sequences were mapped to the human (hg38) and rat (rn6) genomes using HISAT2, with alignment scores calculated from the percentage of aligned reads. Count matrices of gene counts per sample were generated using featureCounts, which were then used to generate graphs of relative gene expression in individual samples.

To further explore the dataset and perform preliminary assessments of DE gene expression and gene ontology (GO) enrichment, a custom Bioconductor-based R script was generated in which initial gene filtering was achieved using the function koverA in the genefilter package. Filtered genes were used as the input to the package DESeq2 to perform size-based normalization and DGE analysis on the data. In this dataset, equal variance across groups was assumed to account for conditions with a sample size of 1. Principal component analysis (PCA), unsupervised clustering, dispersion modeling, and heat mapping of gene expression differences among samples were performed with the complete filtered gene list as well as the differentially expressed genes (DEGs). Further, gene ontology (GO) analysis was performed on sets of upregulated or downregulated genes. A false discovery rate (FDR) of <0.05 was considered statistically significant for both DGE and GO analyses.

### 2.15. Data and Statistical Analysis

RNAseq DEG data was analyzed and graphed in RStudio, and all other data was analyzed and graphed in Prism 9 (GraphPad Software, San Diego, CA, USA). Statistics were calculated using one- or two-way ANOVA as appropriate with Tukey–Kramer post-hoc analysis, with *p*-values < 0.05 considered statistically significant. The data in this study are expressed as the mean ± standard error.

## 3. Results

### 3.1. Medium Composition Influences hiPSC-CM and EC Structure and Function

A primary consideration in fabricating complex, multi-cellular EHMs is the impact of the culture medium on each cell population due to varying levels of ions, growth factors, and other supplements. ECs are maintained in EGM-2, whereas hiPSC-CMs are cultured in RPMI B27, so we sought to evaluate the impacts of mixed medium culture on each cell type individually to determine the most acceptable compromise. We first examined EC and hiPSC-CM viability in 2D culture over 5 days in their standard medium (100/0 or 0/100 CM/EC medium), 70/30 CM/EC mixed medium, or 50/50 medium (Supplementary Figure S1). Despite no significant differences in hiPSC-CM viability, hiPSC-CMs in mixed medium conditions demonstrated markedly increased sarcomere development and hypertrophy relative to hiPSC-CMs in control CM medium (Supplementary Figure S1B,C). This may in part be due to the low presence of factor-rich FBS in EGM-2, which may promote protein production.

**Figure 2:**
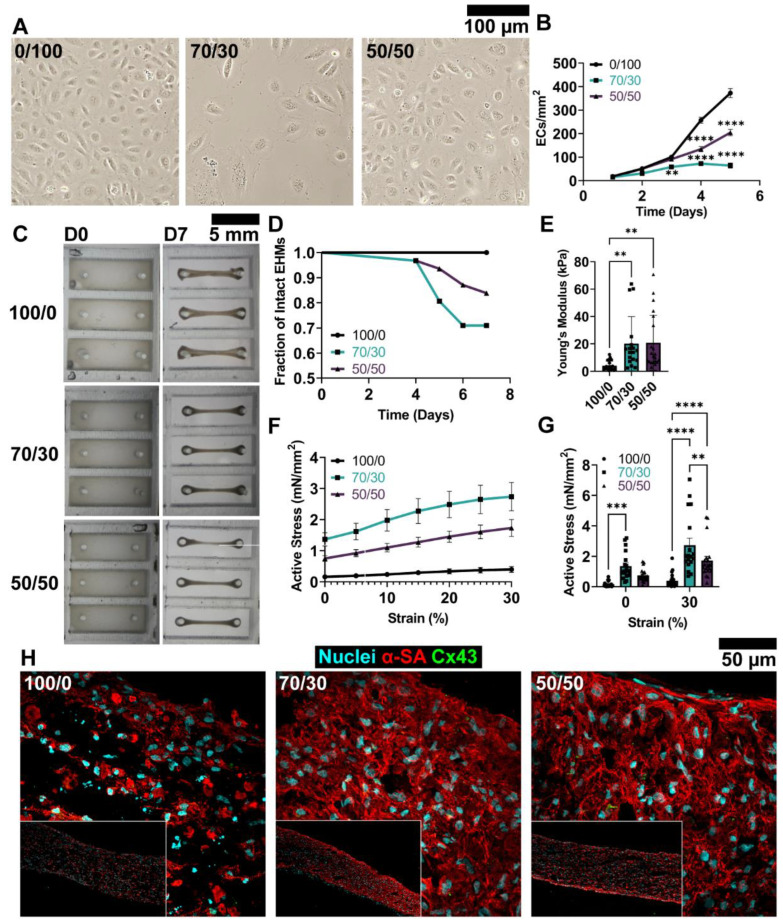
Mixed media culture differentially impacts EC and CM structure and function. (**A**,**B**) EC proliferation and morphology evaluated in monolayer culture with mixed media. *n* = 9 per group. (**C**) Compaction and (**D**) necking of 3D linear EHMs across 7 days of culture. *n* = 18–21 per group. (**E**) Mechanical characterization of passive and (**F**,**G**) active stress generation of EHMs at 5% strain increments up to 30% strain. *n* = 18–21 samples per group. (**H**) Histological staining of EHMs using *α*-SA and Cx43 following mechanical characterization. *α*-SA: *α*-sarcomeric actinin; Cx43: Connexin-43. Mixed media formulations are indicated as percent B27/percent EGM-2 (e.g., 70/30: 70% B27 and 30% EGM-2). ** *p* < 0.01; *** *p* < 0.001; **** *p* < 0.0001.

ECs cultured in 70/30 medium displayed a significant decrease in day 1 attachment relative to control EGM-2, suggesting that medium composition plays a role in EC binding interactions (Supplementary Figure S1A). ECs in the control medium exhibited a characteristic cobblestone morphology and rapidly proliferated until confluence at 5 days (Figure 2A,B). In comparison, ECs cultured in mixed media proliferated markedly slower, resulting in significantly fewer cells present in the wells of the 70/30 condition as early as day 3 (100/0: 100.46 ± 7.39 cells/mm^2^; 70/30: 57.93 ± 5.53 cells/mm^2^, *p* = 0.0036), and showing in the 50/50 condition by day 4 relative to control (100/0: 257.36 ± 12.59 cells/mm^2^; 50/50: 134.37 ± 11.19 cells/mm^2^, *p* < 0.0001). Although ECs in both mixed compositions did not reach confluency within 5 days, ECs in the 70/30 medium proliferated slowly and had qualitatively irregular, spread morphologies. In contrast, ECs in 50/50 medium exhibited primarily a cobblestone morphology in more dense areas, with a few cells adopting a spread morphology.

To evaluate the functional consequences of mixed medium compositions on hiPSC-CMs in 3D culture, we fabricated small (9 × 3 mm) linear EHMs (Figure 2C). We observed increased compaction of EHMs in 70/30 medium relative to 50/50 medium (Supplementary Figure S2A), and particularly many instances of hypercompaction, necking, and failure of the EHMs (*p* = 0.0079 by Mantel-Cox test; Figure 2D). After 7 days of in vitro culture, we performed mechanical analysis on the tissues, followed immediately by fixation and histological analysis. It is important to note that mechanical evaluation was performed only on intact tissues and thus may underestimate the true mechanics of groups that experienced greater compaction and necking. Tissues exhibited significantly greater stiffness (Figure 2E) in mixed media culture (70/30: 20.18 ± 4.67 kPa, *p* = 0.0071; 50/50: 20.10 ± 4.39 kPa, *p* = 0.0033) relative to 100/0 control tissues (4.08 ± 0.71 kPa). Active twitch stress was also significantly different across groups (Figure 2F,G), with 70/30 tissues generating greater twitch stress (1.37 ± 0.21 mN/mm^2^, *p* = 0.0006) relative to control tissues (0.16 ± 0.04 mN/mm^2^) at 0% strain, and both mixed media groups (70/30: 2.74 ± 0.46 mN/mm^2^, *p* < 0.0001; 50/50: 1.73 ± 0.27 mN/mm^2^, *p* < 0.0001) outperforming control tissues (0.40 ± 0.1 mN/mm^2^) at 30% strain, in line with morphological observations of increased myofilament development and hypertrophy. We consequently also observed similar significant increases in upstroke velocity, T50, and T90 (Supplementary Figure S2B–D). Lastly, we performed force-frequency response analysis and noted that irrespective of condition, all tissues demonstrated an inverse relationship between pacing frequency and force generation (Supplementary Figure S2E) and were able to follow pacing up to 4 Hz (Supplementary Figure S2F). During the force-frequency response protocol, tissues began exhibiting contractile alternans at 2 Hz, which increased in prevalence with pacing frequency regardless of condition (Supplementary Figure S2G). Calculating relative amplitude compared to full contraction trended towards more severe partial-force contractions in mixed media conditions, which reached significance at 4 Hz between 70/30 (78.7 ± 6.3%, *p* = 0.0035) and control (91.2 ± 2.5%; Supplementary Figure S2H) [46]. Lastly, immunohistochemical staining of tissues following mechanics analysis revealed noticeable improvements in sarcomere development and mild hypertrophy, corroborating 2D histological data, with the greatest sarcomere density in the 70/30 medium group (Figure 2H).

Together, these results indicate that a mixed medium composition of RPMI B27 and EGM-2 improves hiPSC-CMs contractility likely through increased sarcomere development but negatively impacts EC viability and proliferation. The 70/30 ratio best improves EHM mechanics but consequently increases the relative severity of contractile alternans. This formulation also negatively impacts the morphology and proliferation of ECs, which could compromise proper vessel formation and function. Thus, we selected the 50/50 medium mixture for all further experiments.

### 3.2. Coating Solution Enables Local Delivery of ECs to Channel Wall

**Figure 3:**
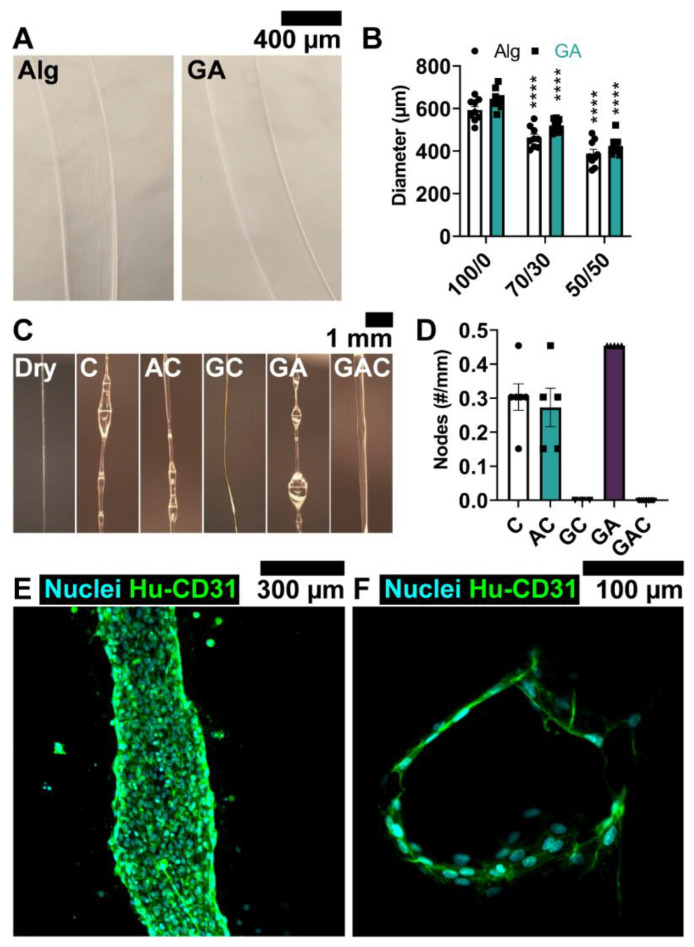
Formation of patterned vasculature using EC-coated gelatin-alginate fibers. (**A**,**B**) Equilibrium swelling and diameter assessment of gelatin-alginate fibers in mixed media culture. *n* = 9 per group. Only significance relative to 100/0 medium of the same fiber composition shown. (**C**,**D**) Coating solutions composed of gelatin, alginate, and/or collagen used to evaluate homogeneity in EC seeding. *n* = 3 per group. (**E**) EC-coated vessel using gelatin-alginate-collagen solution in whole mount and (**F**) transverse sections. C: Collagen; A: Alginate; G: Gelatin; Hu-CD31: Human-specific CD31. Mixed media formulations are indicated as percent B27/percent EGM-2 (e.g., 70/30: 70% B27 and 30% EGM-2). **** *p* < 0.0001.

We modified our previously published alginate fiber wet spinning technique [47] by incorporating 2.5% *w*/*v* gelatin into the 100 mM CaCl_2_ crosslinking bath, generating sacrificial alginate fibers ensheathed in gelatin (GA fibers) to provide a surface for EC attachment. In considering the co-culture application of these fibers, we quantified diameter by incubating the fibers in 100/0, 70/30, and 50/50 media (Figure 3A). Image analysis showed an insignificant diameter increase when alginate fibers were coated in gelatin, consistent with a thin coating. However, an inverse relationship between EGM-2 content in medium and fiber diameter was observed irrespective of fiber composition, with 50/50 medium resulting in the smallest diameter GA fibers (GA 50/50: 425 ± 14.27 μm, *p* < 0.0001) relative to both 70/30 (GA 70/30: 520.05 ± 10.08 μm, *p* = 0.0003) and control media (GA 100/0: 643.06 ± 15.52 μm; Figure 3B). This pattern was present in alginate fibers as well. The decrease in fiber diameter is likely due to a greater divalent cation concentration in mixed media formulations, including calcium (~1.6 mM in basal EBM-2, compared to 0.42 mM in RPMI 1640), which is involved in crosslinking alginate chains, although additional protein content contributions from FBS in EGM-2 may also play a role in equilibrium swelling.

We next developed a coating solution to homogeneously seed ECs on the outer surface of the GA fibers. Fibers were positioned by embedding them into the walls of PDMS molds to replicate the final EHM-casting protocol, and a variety of coating solutions were tested. A viscous hydrogel-based solution was necessary because seeding endothelial cells in culture medium alone resulted in the suspension dripping off the fibers before the cells adhered. Seeding in a 1 mg/mL collagen solution was uneven and tended to ball up in areas forming nodes, likely due to self-cohesion and a slow gelation time for the coating. With iteration, we found that a multicomponent coating solution of 1% *w*/*v* gelatin, 0.25% *w*/*v* alginate, and 1 mg/mL collagen enabled facile and uniform coating along the length of the fiber using a micropipette (Figure 3C). The inclusion of gelatin was necessary to reliably create a smooth interface with GA fibers and minimize node formation, while a low concentration of alginate provided rapid crosslinking with residual calcium on the GA fibers prior to longer-term gelation of the collagen to form a stable gel layer (Figure 3D). We tested the optimized coating solution in vitro by incorporating ECs at a density of 2.5 × 10^7^ cells/mL in the coating solution, applying the seeding solution to the fibers (approximately 1.25 × 10^5^ ECs total), and embedding the coated GA fibers in a 2 mg/mL collagen gel. GA fibers were un-crosslinked and washed out the next day to create patent vessels. Culture over 5 days in 50/50 medium resulted in nearly full circumferential coverage (Figure 3E,F) and conserved patency, demonstrating successful EC delivery and engineered vessel formation with patency maintained for at least 5 days in vitro.

### 3.3. Dynamic Perfusion Culture of vEHMs

In order to develop larger constructs with perfusable vasculature for therapeutic effect in rats, we scaled up our fabrication process to create 10 × 12 mm tissues (prior to compaction). The PDMS molds included a thicker wall section on one end to accommodate the insertion of a stainless steel needle for dynamic perfusion culture of vEHMs. GA fibers were embedded into the walls of the mold to create an array pattern of intersecting channels, and bundled collagen microfibers were embedded along the longitudinal margins of the tissue mold to provide greater structure to the tissue, improved handleability during implant, and height alignment with the perfusion needle (Supplementary Figure S3).

**Figure 4:**
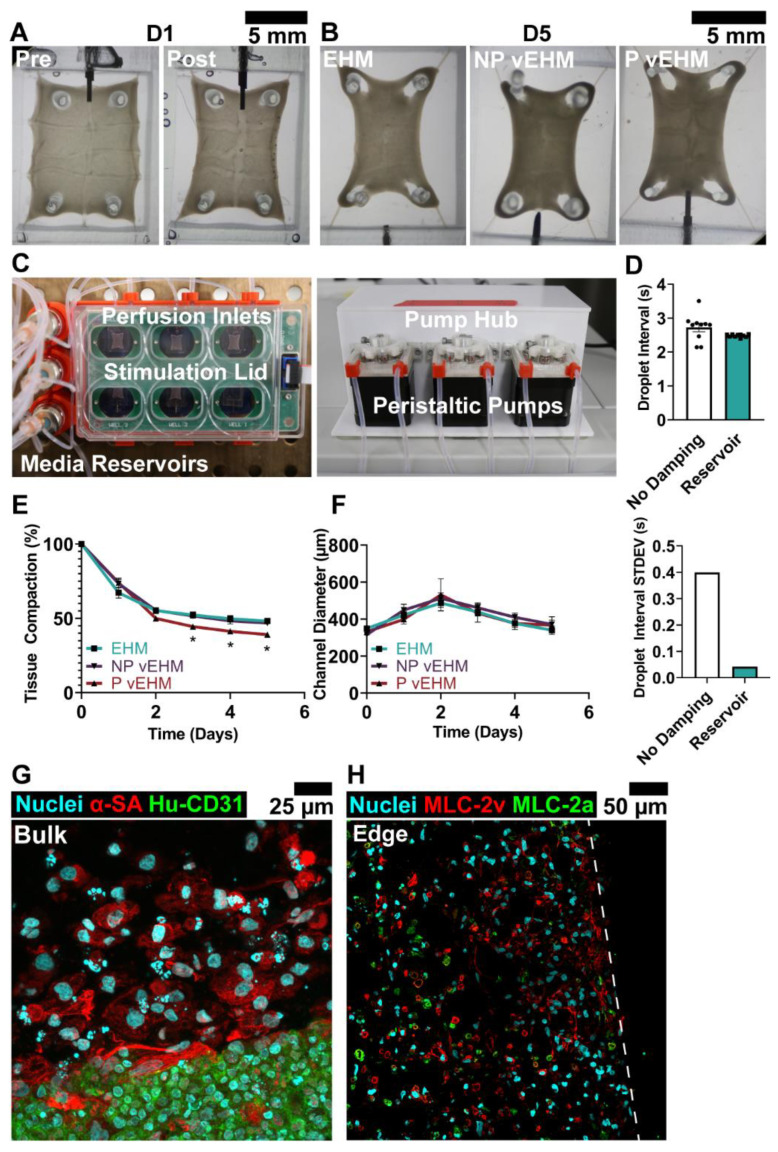
Fabrication and perfusion culture of vEHMs. (**A**) Fabricated EHMs and vEHMs before and after treatment with un-crosslinking solution. (**B**) EHMs and vEHMs prior to implantation after 5 days of culture. (**C**,**D**) P vEHMs are perfused using a custom peristaltic pump system. (**E**,**F**) Tissue compaction and channel diameter over culture period. *n* = 11–14 per group. (**G**) Histological evaluation of vEHMs adjacent to a patterned vessel and (**H**) near the tissue edge (dotted line). *α*-SA: *α*-sarcomeric actinin; Hu-CD31: Human-specific CD31; MLC-2v: Myosin light chain 2v; MLC-2a: Myosin light chain 2a. * *p* < 0.05.

To form tissues, GA fibers were coated with ECs (2.5 × 10^7^ cells/mL; vEHMs) or without ECs (control EHMs) prior to casting a cell-collagen mixture containing hiPSC-CMs (1.5 × 10^6^ cells/mL) with 5% hCFs and incubating overnight in 50/50 medium. The next morning, GA fibers were un-crosslinked, and residual alginate was washed out to establish patent vessels (Figure 4A,B). After chelation, a subset of tissues with endothelialized vessels were connected to a peristaltic pump-based perfusion system (Figure 4C) for open loop recirculation of medium (P vEHMs) or not perfused (NP vEHMs), and all tissue conditions were paced under 1 Hz field stimulation (Video S2). A reservoir system was incorporated into the flow circuit to dampen the natural pulsatile flow of the pump (Figure 4D) and trap air bubbles to prevent embolism. The fluid velocity of the pump was determined by the rotational speed of the connected stepper motors, which were controlled by an Arduino. Peristaltic pump flow was experimentally measured as 7.28 mL/h, and average wall shear stress was calculated to be 3.21 dyn/cm^2^ assuming Poiseuille flow and an average vessel diameter of 400 μm, which is a low yet physiologically relevant shear stress for ECs [56,57] and within the reasonable range (1–10 dyn/cm^2^) of comparable literature [13,58,59,60].

During 5 days of in vitro culture, significant remodeling occurred, with P vEHMs compacting significantly more (39.08 ± 1.83%) than non-vascularized control EHMs (48.24 ± 1.80%, *p* = 0.014) or NP vEHMs (46.89 ± 1.76%, *p* = 0.038; Figure 4E). Diameter increased transiently during this time due to the un-crosslinking treatment of fibers, which caused swelling of the alginate chains with calcium chelation prior to washout. Fiber diameter re-stabilized to approximately 400 μm after GA fiber washout and remained similar across groups (Figure 4F). Histological evaluation at 5 days revealed endothelialized vessels in vEHMs, with elongated hiPSC-CMs primarily located at the edges of tissues and more rounded cardiomyocytes located in the bulk of tissues where there was a lack of passive tension cues to direct alignment (Figure 4G,H). These results demonstrate that robust vEHMs with stable, perfusable vasculature can be fabricated and cultured in vitro.

### 3.4. P vEHMs Exhibit Semi-Conserved Pattern Morphology and Improved Engraftment

**Figure 5:**
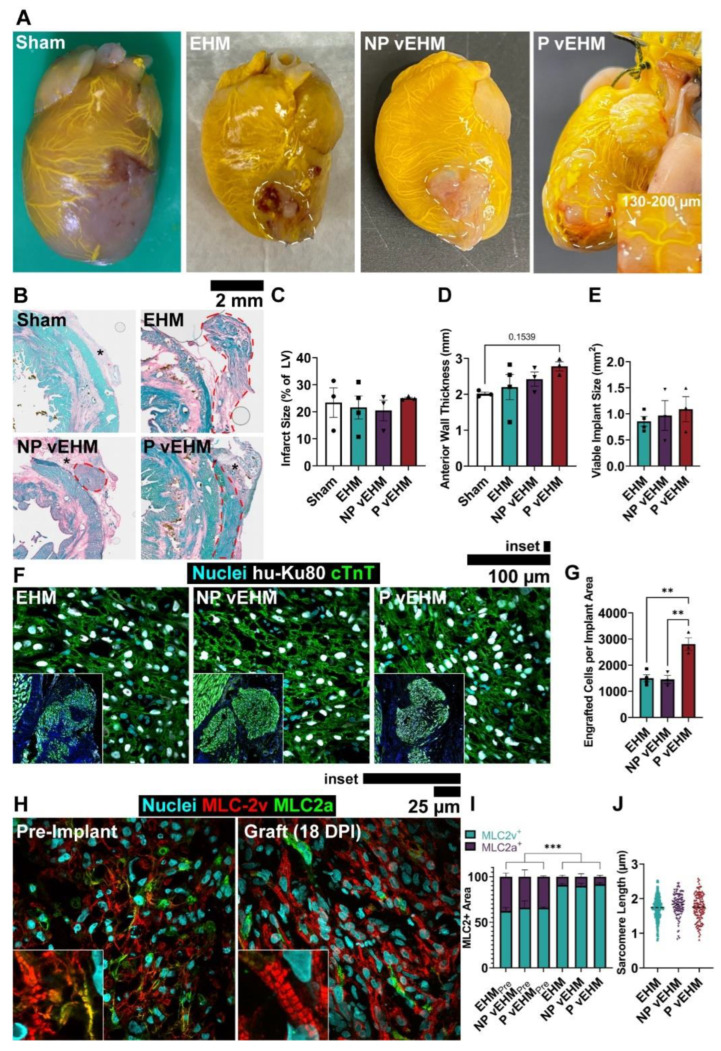
Implants mature in vivo, and engraftment is best improved by in vitro perfusion. (**A**) Explanted hearts after perfusion with radiopaque dye Microfil^®^. Dotted lines indicate implants. Inset: Vasculature in P vEHM with preserved array geometry. (**B**) Picrosirius red—fast green staining of transverse heart sections showing anterior free wall with infarct and implant (red dotted lines). Asterisks indicate adhesion tissue. (**C**) Quantification of infarct size, (**D**) anterior wall thickness excluding implants, and (**E**) viable implant size. (**F**,**G**) Histological evaluation of human cell engraftment with Hu-Ku80 staining. (**H**,**I**) Histological evaluation of graft maturation with atrial and ventricular myosin light chain isoforms. (**J**) Quantification of hiPSC-CM sarcomere length in implants. *n* = 3–4 per group. Hu-Ku80: Human-specific Ku80; cTnT: cardiac troponin T; MLC-2v: Myosin light chain 2v; MLC-2a: Myosin light chain 2a; DPI: Days post-injury. * *p* < 0.05; ** *p* < 0.01; *** *p* < 0.001.

Following in vitro fabrication and assessment of vEHMs, we evaluated engraftment onto infarcted hearts using a rat model of ischemia-reperfusion MI. EHMs were implanted 4 days after the infarct, and cardiac function was evaluated after two weeks, with two animals excluded from further analysis due to small infarcts that did not impact FS. No group (*n* = 3–4/group) displayed significant improvements in heart function from 1D M-mode echocardiography images by calculated FS and ejection fraction (EF) at the 2-week time point despite promising trends for the P vEHM group (Supplementary Figure S4); however, this result is neither surprising nor unexpected based on the relatively short study duration, which was selected to evaluate early yet persistent neovascularization rather than contractile function. Hearts were then harvested and underwent ex vivo OCT and microCT imaging prior to sectioning for histological evaluation. During Microfil^®^ perfusion, we observed a distinct vessel array reminiscent of the original pattern geometry in 1 of 3 P vEHM implants (Figure 5A). Further, EHM and NP vEHM implants were distinctly paler in color than P vEHMs, suggesting greater intra-implant perfusion in the latter group.

Morphometric analysis of hearts from picrosirius red—fast green stains indicated comparable infarct size and anterior wall thickness excluding implants (Figure 5B–D). Implants were clearly visible (Figure 5B, red dotted lines) and often surrounded by vascularized adhesion tissue from the chest wall (Figure 5B, asterisks). Viable implant size was quantified by measuring the area of live hiPSC-CMs from immunohistochemical stains of Hu-Ku80 and cTnT, but no significant difference was found between groups (EHM: 0.86 ± 0.09 mm^2^, NP vEHM: 0.97 ± 0.29 mm^2^, P vEHM: 1.09 ± 0.24 mm^2^; Figure 5E,F, Supplementary Figure S5A).

However, nuclei quantification revealed significantly greater human cell density in P vEHMs (Supplementary Figure S5B) as well as increased engraftment when normalized by implant area (2806 ± 239.3 Hu-Ku80+ nuclei/mm^2^) as compared to EHMs (1499 ± 132.0 Hu-Ku80+ nuclei/mm^2^, *p* = 0.0024) and NP vEHMs (1452 ± 154.1 Hu-Ku80+ nuclei/mm^2^, *p* = 0.0029; Figure 5F,G). There was no notable fragmentation of Hu-Ku80+ nuclei in any group that would be suggestive of ongoing implant apoptosis or necrosis. Total intra-implant cell nuclei were not different across groups (Supplementary Figure S5C), but evaluation of co-stained cTnT+ area in implants showed trends towards increased hiPSC-CM area in P vEHMs (22.01 ± 1.68%) that did not reach significance relative to NP vEHMs (14.41 ± 2.19%, *p* = 0.13) or control EHMs (15.75 ± 2.03%, *p* = 0.089; Supplementary Figure S5D), although this metric may be confounded by relative hiPSC-CM compaction and remodeling with the host across groups. We then assessed hiPSC-CM proliferation by Hu-Ki67 staining (Supplementary Figure S5E,F) and found no differences across groups. Together, these results suggest that the difference in engraftment of P vEHMs is primarily due to the greater survival of hiPSC-CMs (rather than proliferation of hiPSC-CMs, engraftment of ECs and hCFs, or infiltration of host cells).

Ventricular isoform expression of MLC-2 was greater in all implant groups (89.68–91.65% MLC-2v+ by area) compared to in vitro pre-implantation controls (62.16–65.75% MLC-2v+ by area, all *p* < 0.001), although there were no differences between implant groups (Figure 5H,I). Similarly, measurements of sarcomere length in implants were not significantly different between implant groups (EHM: 1.73 ± 0.02 μm, NP vEHM: 1.82 ± 0.03 μm, P vEHM: 1.75 ± 0.03 μm; Figure 5J), indicating that engraftment promoted maturation as assessed by MLC-2v isoform switching for all tissues. Together, these data provide evidence that perfused, patterned engineered vessels can be conserved and perfused in vivo by inosculation, and that in vitro perfusion of patterned vessels improves hiPSC-CM survival and engraftment density.

### 3.5. P vEHMs Develop Larger and More Hierarchically Organized Vasculature

**Figure 6:**
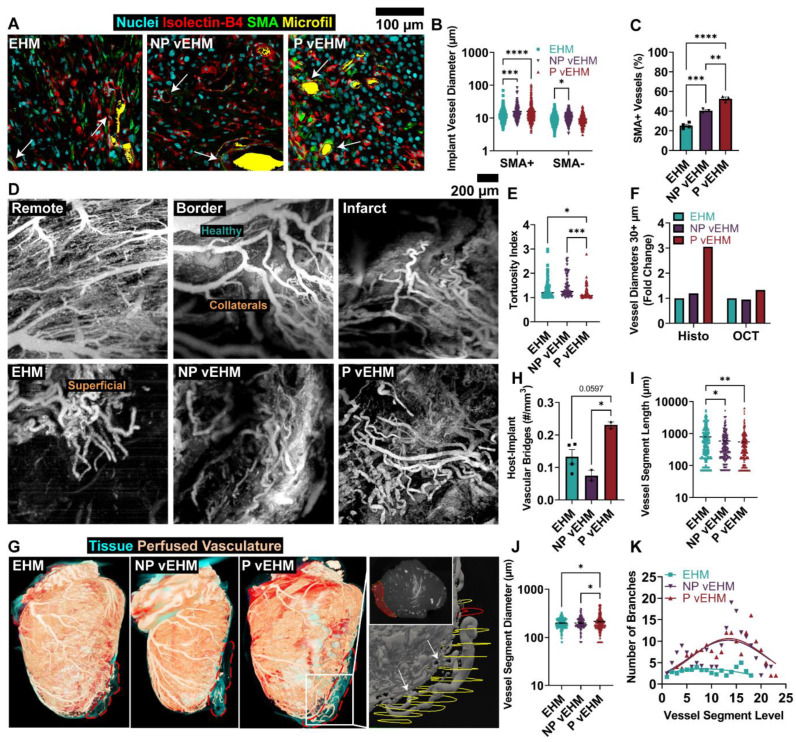
Patterned vasculature increases implant vessel diameter, but in vitro perfusion best improves implant vessel organization. (**A**) Representative histological images of intra-implant vessels and supportive stromal cells (white arrows). (**B**) Diameter distribution for vessels with (SMA+) or without (SMA-) SMA expression. (**C**) Percent of SMA-expressing vessels in implants. *n* = 3–4 per group. (**D**) Representative images of perfused vasculature imaged with OCT in the remote, border, infarct, and implant regions. (**E**) Tortuosity index and (**F**) intra-implant diameter of perfused vasculature imaged with OCT. *n* = 3–4 per group. (**G**) MicroCT scans of cardiac and intra-implant vasculature (red-white) overlaid on tissue structure (teal). Red dotted lines indicate implants. Magnified image (right) shows implant vasculature encased in implant segmentation (yellow contours) at successive cross-sections (inset, red area corresponding to red contour). Quantification of (**H**) host-implant vascular bridges and intra-implant vessel segment (**I**) length, (**J**) diameter, and (**K**) branching per segment level. *n* = 2–3 per group. SMA: Smooth muscle actin. OCT: Optical coherence tomography. * *p* < 0.05; ** *p* < 0.01; *** *p* < 0.001; **** *p* < 0.0001.

To identify how perfused, patterned vessels in EHMs affect vascular development in vivo, we first performed histological quantification of pan-vascular isolectin-B4 and Hu-Ku80 in the remote, infarct, and implant regions of each group, although no significant differences were found (Supplementary Figure S6A–C). We next performed histological stains for isolectin-B4 and SMA (cross-registered to Hu-Ku80 stains to only quantify vessels in areas of viable implant) and found significantly increased diameters of SMA-expressing (SMA+) vessels in NP (15.89 ± 0.72 μm, *p* = 0.0004) and P vEHMs (16.06 ± 0.71 μm, *p* < 0.0001) compared to control EHMs (13.16 ± 0.52 μm; Figure 6A,B). This was accompanied by a greater frequency of SMA+ vessels in P vEHMs (52.69 ± 1.66%, *p* < 0.0001 relative to EHM and *p* = 0.0017 relative to NP vEHM) and, to a lesser extent, in NP vEHMs (40.46 ± 1.06%, *p* = 0.0003) as compared to EHMs (25.31 ± 1.41%; Figure 6C), suggesting greater in vivo arteriogenesis with in vitro perfusion. Co-staining for human-specific CD31 (Hu-CD31) with isolectin B4 revealed two distinct phenotypes in NP and P vEHMs. Human ECs either appeared in capillary-like sprouts in NP vEHMs (Supplementary Figure S6D) or were associated with vessel walls, potentially indicating a patterned vessel (Supplementary Figure S6E). This latter phenotype was exclusively present in P vEHMs, as no isolated human EC sprouts were found. Further, while Microfil^®^ overlays were captured via light microscopy where possible, the material was not reliably maintained in vessels (particularly those with larger lumens) during histological processing and thus could not be used as an accurate metric for perfusion. However, some Microfil^®^-filled lumens did contain Hu-CD31+ cells in NP and P vEHMs, providing evidence of functional inosculation and perfusion of patterned vessels in both groups and/or evidence of human ECs incorporating into newly formed vessels of rat origin.

To better understand the 3D architecture and perfusability of intra-implant vasculature, we performed OCT imaging of the remote, infarct, and implant regions for each group (Figure 6D, Supplementary Figure S7A–C). Cardiac microangiograms were acquired using a 20% Intralipid solution as contrast to visualize hierarchically branched vascular architecture down to the capillary scale. Acquisition at border and infarct regions displayed increasingly tortuous vasculature and collaterals and less resolvable capillary perfusion. EHM microangiograms exhibited variable perfusability, often with gaps that appeared lowly perfused and/or highly tortuous (1.35 ± 0.04 tortuosity index). In contrast, microangiograms of vEHMs revealed greater perfusable vessels with typically larger sizes and, notably, significantly less tortuosity in P vEHMs (1.2 ± 0.03 tortuosity index) relative to NP vEHMs (1.46 ± 0.06 tortuosity index, *p* = 0.0002) and control EHMs (*p* = 0.025; Figure 6E). We quantified diameter from OCT microangiograms and, akin to histological assessments, found trends of larger vessels in NP and P vEHMs (Figure 6F, Supplementary Figure S7D,E), although this did not reach significance relative to control.

Following OCT microangiography, the hearts were perfused with Microfil^®^ to enable microCT-assisted reconstructions of the cardiac vasculature (Figure 6G, Video S3–S5). Three hearts were excluded from further analysis due to poor Microfil^®^ perfusion. The implant regions were isolated for vascular reconstructive analysis by manual segmentation and cross-registration with histological sections. We quantified host-implant vascular bridges and observed a significant increase in P vEHMs (0.231 ± 0.010 bridges/mm^3^) relative to NP vEHMs (0.075 ± 0.016 bridges/mm^3^, *p* = 0.018) and a trending increase relative to control EHMs (0.133 ± 0.022 bridges/mm^3^, *p* = 0.059) that did not quite reach significance (Figure 6H). Total intra-implant vascular volume did not reach significance across groups (Supplementary Figure S7F), and there was significantly decreased vessel segment length in endothelialized vEHMs (NP vEHM: 583.6 ± 47.09 μm, *p* = 0.036; P vEHM: 546.2 ± 48.98 μm, *p* = 0.007) relative to control EHMs (787.6 ± 72.39 μm; Figure 6I). In contrast, vessel segment diameter was significantly increased in P vEHMs (213.7 ± 5.61 μm) relative to NP vEHMs (196.6 ± 4.01 μm, *p* = 0.024) and EHMs (196.5 ± 3.47 μm, *p* = 0.039; Figure 6J). We also observed trends towards increased branching per segment level in endothelialized vEHMs (Figure 6K, Supplementary Figure S7G), pointing to greater vascular hierarchy than EHM. Together, these results demonstrate that patterned vasculature enables the development and maintenance of stable, large-diameter vessels with greater branching than control tissues, and in vitro perfusion enables a more stable EC phenotype with less human EC migration away from patterned vessels in vivo and greater hierarchical organization.

### 3.6. Exploratory Bulk RNA Sequencing of Explanted vEHMs Suggests Perfusion Influences hiPSC-CM Development and Contractility

We sought to further understand the effects of implantation and patterned, perfused vasculature on hiPSC-CM genotype (as a precursor to phenotype shifts) through exploratory bulk RNAseq analysis of explanted tissues. At the two-week timepoint, some implanted tissues were manually debrided from the epicardial surface and processed for bulk RNA isolation and sequencing. Alignment analysis produced the expected heterogeneous mixture of human and rat-derived reads in explanted tissues and, by comparison, almost 100% human-aligned reads in in vitro control tissues (Supplementary Figure S8A). Notably, two samples had uncharacteristically low alignment scores with the human genome and were thus excluded from further analysis. Principle component analysis plots showed stratification by condition, with implants expectedly segregated from in vitro control tissues primarily by principal component 1 (representing 49.2% of differences) due to contributions of the local in vivo microenvironment, and implant groups further separated across principal component 2 (representing an additional 19.1% of differences, Supplementary Figure S8B). Normalized counts for genes of interest in cardiomyocyte development, maturation, and vascularization were plotted for each condition, revealing trends of upregulation of developmentally regulated genes involved in myofibril assembly and cardiac contractility (e.g., MYH1, MYH3, MEF2C, XIRP2, MYL2, MYL7) and calcium handling (e.g., ATP2A1, ATP2A2; Supplementary Figure S8C–I). Vascularization panels for human ECs showed trends for downregulation of several growth factors and receptors relative to in vitro non-perfused controls (e.g., VEGFA, FGF11, IGF1R, PDGFRA, PDGFRB, FLT1), suggesting reduced human vascular signaling in vivo that is consistent with the turnover of human ECs for host rat ECs (Supplementary Figure S8F,G).

To further explore differential regulation between groups, we performed a preliminary analysis of DEGs between explant groups, assuming equal sample variance to account for poor sample sizes owing to samples excluded due to low alignment to the human genome (final *n* = 1–2/group). In this analysis, only 9 and 13 genes were identified as differentially expressed for P vEHM vs. EHM and P vs. NP vEHM, respectively. Subsequent GO analysis produced terms involved in myofilament development, calcium handling, and contractility, implying a role for in vitro perfusion of vEHMs to amplify the in vivo development of hiPSC-CMs. However, the sample size and subsequent methodology, small numbers of DEGs, and low expression levels of some genes dampen the impact of these preliminary findings. Our RNAseq results implicate a role for vascularization and perfusion in the biological development of hiPSC-CMs, providing some support for our hypothesis that a co-vascularization strategy for remuscularization by implanted vEHMs is beneficial for holistic and robust cardiac regeneration.

## 4. Discussion

Vascularization remains a primary concern when developing implantable cardiac tissues for optimal engraftment and integration, and further, it is unclear what the impact of vasculature is on hiPSC-CM survival, integration, development, and maturation in vivo. In this study, we demonstrate a facile method for fabricating and culturing EHMs with patterned vasculature under static or dynamic perfusion conditions. Our results indicate that in vitro-perfused patterned vessels in vEHMs best improve in vivo hiPSC-CM survival and engraftment density, implant vascular organization and perfusion, and potentially also hiPSC-CM contractile development, as suggested by RNAseq analysis. Compared to other vessel patterning techniques, which require expensive equipment and/or materials that cannot be autoclaved or otherwise easily sterilized [10,11], our methodology provides a flexible foundation for creating perfusable and implantable vEHMs in standard 6 well tissue culture plates and uses equipment and biomaterials that can easily be fabricated in-house using our open-source plans or otherwise commercially available materials and technologies. Our results are the first to directly support the use of perfused, endothelialized vessels in engineered human cardiac implants to improve both the vascular bed and the engraftment of hiPSC-CMs.

The success of hiPSC-CM engraftment after EHM implantation onto the infarcted LV is a function of multiple design parameters. The relatively immature metabolic profile of terminally differentiated hiPSC-CMs may facilitate survival due to their greater resistance to ischemia, such as that experienced at implantation [33]. Although the CM/EC media tested in this study are devoid of fatty acids and thus do not facilitate metabolic maturation, the selected 50/50 culture medium enabled some structural maturation of CMs (Figure 2H, Supplementary Figure S1C) [24,29], which could be due to increased calcium in the growth medium (EBM-2 basal media contains ~1.6 mM Ca) during in vitro culture [61] as well as the presence of growth factors including VEGF [2,62,63] and IGF [34,49,64]. The greatest improvements in hiPSC-CM engraftment were observed in P vEHMs, indicating that the presence of ECs in the channels alone (NP vEHMs) was not sufficient to maintain hiPSC-CM density upon implantation but could be rescued by in vitro perfusion. This metric of hiPSC-CM engraftment density was higher than comparable studies despite similar graft sizes [2,12,13,65,66] and was independent of proliferation (Figure 5G, Supplementary Figure S5). Studies on cardiac function, and particularly the influence of patterned vessels on engrafted hiPSC-CM maturation at longer timepoints, are critical for advancing this therapeutic approach in the clinic. We hypothesize that shear stress on ECs modulates their phenotype, likely also altering EC-CM crosstalk, to improve inosculation and engraftment of the implanted tissue, which will be pursued in future work.

The burning question to explore is what enables patterned vessels to be functionally utilized by the regenerating vasculature through the process of inosculation. Not all patterned vessels could be reliably identified owing to remodeling, EC turnover, and the limitations of cardiac vascular imaging. However, evidence of residual Microfil^®^ in Hu-CD31+ vessels of NP and P vEHMs (Supplementary Figure S6D,E) suggests that channel endothelialization facilitates inosculation and perfusion of patterned vessels and that up-stream arteriogenic processes may be required of host vasculature to facilitate greater blood flow across the vascular bridges from host to implant. In vitro perfusion may also have facilitated a more stable EC phenotype that was less prone to migration away from patterned channels when implanted in vivo, as evidenced by the lack of isolated human ECs in P vEHMs compared to NP vEHMs. Further, histological staining showed greater numbers of SMA+ vessels in P vEHMs of all diameters compared to other groups (Figure 6A–C). This indicates endothelialization and particularly in vitro perfusion can facilitate the process of vessel stabilization and maturation of both inosculated vessels and angiogenic branches by recruitment of SMA-expressing stromal cells (such as smooth muscle cells, fibroblasts, and pericytes), ultimately resulting in faster and more stable implant perfusion post-implantation. This is supported by a recent study by Brady et al., which showed that their (non-perfused) patterned microvessels were disrupted when implanted on the rat heart, resulting in isolated ECs reminiscent of one of the vascular phenotypes of human ECs seen in NP vEHMs [67]. Further, single-cell and bulk RNAseq experiments of ECs exposed to flow showed differential regulation of genes, suggesting a more quiescent and stable phenotype than no-flow controls [68]. Inosculation is further evidenced by the conserved pattern geometry visually confirmed in one out of three vEHM-implanted hearts perfused with Microfil^®^ (Figure 5A, right, inset). It remains unclear whether inosculation occurred in the other two vEHM-implanted hearts, as macroscopic visual assessment and microCT reconstructions were not conclusive, despite the presence of Microfil^®^ within Hu-CD31-positive vessels by 2D immunohistochemical analysis. Inosculation of some vessels may have been obscured by in vivo remodeling of human EC-lined channels into perfusable vessels or by unresolved thrombosis resulting in partial perfusion of the patterned vessel array [69,70].

Other studies have reported that larger-diameter endothelialized vessels (>400 μm) can facilitate vessel retention, inosculation, and perfusion in vivo, although often with remodeling to a smaller diameter [9,58,71]. While smaller diameter microvessels (~50–200 μm) also show variable success in metrics of inosculation and perfusion [8,12,13,67,72], direct comparison of 200 μm and 400 μm patterned vessels in a study by Mirabella et al. demonstrated that the smaller vessels were unable to rescue perfusion in a hind-limb ischemia model, indicating initial patterning diameter remains an important consideration in revascularization [9]. The presence of channels alone without ECs (as in control EHMs) can directionally instruct host vessel ingrowth and thus provide an indirect in vivo benefit in revascularization [73,74,75], although we were unable to reliably and definitively identify EHM channels due to remodeling to confirm this phenomenon in our study. It remains unclear whether this can provide functional therapeutic benefit, as groups including Mirabella et al. showed no improvements in perfusion of the hind limbs with non-endothelialized channels [9]. Similarly, Szklanny et al. showed that non-endothelialized channels (1500 μm diameter) were incapable of effective long-term perfusion even when directly anastomosed to the femoral artery of rats and even reported some animal mortality in this group, hypothesized to be from a thrombus that formed in the channel and dislodged. This further supports the idea that non-endothelialized channels are not viewed by the host as true vessel lumens for inosculation and at best act via indirect mechanisms to guide angiogenic sprouts [71].

In stark contrast to patterning methodologies, reliance on vascular self-assembly via bulk seeding of ECs alone seemingly provides little inosculative perfusion [13] or penetration into implanted tissue [72] without co-seeding with stromal cells [7,69] or in combination with patterned vessels of any scale [13,58]. Our previous work demonstrated significant benefits for vessel development from angiogenic growth factor release from microspheres embedded in hiPSC-CM implants [2], suggesting that engineered paracrine signaling may circumvent the need for bulk-embedded ECs. Together, these results motivate future experiments to explore a dual microvascular and meso-vascular approach to implant revascularization using growth factor release and arteriole-scale vessels and to evaluate how autocrine and paracrine EC signaling both within implants and between host and implant ECs differentially modulate new vessel formation.

To assess small vessel perfusion in a thicker volume of tissue, OCT imaging provides a state-of-the-art imaging modality that can be applied to the heart ex vivo. OCT is not widely available in preclinical studies, and few other groups have performed OCT imaging of the heart [18] and of implanted vascular tissues [13]. To our knowledge, only one other group has published OCT images of implanted cardiac tissue as proof-of-concept that patent vessels can be imaged [12]. The high fidelity microangiograms achieved in this study allow for quantitative comparisons across implant groups, and we demonstrated that greater in vivo vessel organization could be achieved by in vitro perfusion of patterned vessels to avoid tortuous vessel development reminiscent of collateral vessels in the infarct and border regions (Figure 6D,E). Capillary level perfusion is particularly difficult to image in the deeper injury region of the LV wall and epicardial implant and often complicated due to artifacts of the fixed vessels (with reduced compliance), perfusion pressures, capillary collapse, adhesion tissue leakage from the explant, operator skill, or other reasons, making quantification of flow rate and vascular resistance very difficult in these studies. Despite these challenges, future work to examine intra-implant perfusion velocity using dynamic light scattering OCT [76] and longitudinal OCT acquisition should be pursued to advance our understanding of blood flow dynamics in the implant and infarct regions of the post-MI regenerated heart.

As vascular regeneration aims to support larger and thicker tissues in translational applications, engineering the vascular network hierarchy requires the development of whole-implant and even whole-organ quantitative vessel analyses in three dimensions. Our group has previously published on the use of microCT for 3D vascular reconstruction and analysis [2]. In this study, we implemented a vessel reconnection algorithm based on the minimum-work principle [53] to semi-automate the processing steps and provide more accurate metrics of the vessels in implanted tissues. As a result, this advanced analysis provides greater insight into the intra-implant vessel hierarchy. Despite no differences in lumen density across conditions in either the infarct or implant regions (Supplementary Figure S6B) in our histological assessment, we saw an increased frequency of larger-diameter vessels in vEHMs relative to capillary-scale vasculature (Figure 6B). Our microCT analysis of vessel diameter corroborated histological measurements and further revealed greater branching and, interestingly, more host-to-implant vessel bridges in P vEHMs (Figure 6G–K). Together this suggested that a more hierarchical vascular bed formed with endothelialized vessels, and was most functionally improved by in vitro perfusion of vEHMs.

Due to the large data sets generated with whole heart reconstruction that make processing computationally costly, we analyzed only the implant volume at a scan resolution of 50 μm to capture large vessels and meso-diameter arterioles. An artifact of this methodology was a number of single-voxel “vessels” with a reported diameter and length of 50 μm, which were eliminated from our analysis to provide a more accurate quantification of vessel hierarchy. The resulting branching analysis (Figure 6K) shows a negative parabolic relationship between vessel segment level and the number of branches. While expected from a native vascular network of arteries that branch into capillaries and coalesce back into veins, the resolution limits of our analysis suggest this relationship is rather an artifact of branch “termination” below 50 μm in diameter. Use of the highest possible scanning resolution (5–10 μm) requires both sub-sampling implant volume and optimization of Microfil^®^ perfusion to ensure the greatest possible perfusion of the vascular tree, although our vascular volume (Supplementary Figure S7B) is comparable to published best-performing protocols in skeletal [77] and cardiac [17] muscle. The presence of intra-implant vasculature visualized by OCT and microCT data as well as vascular bridges indicates appropriate perfusion through implants. Identification of arterial and venous vasculature to assess relative perfusion efficiency may be pursued in the future by co-registration of OCT microangiograms and velocity maps with microCT data sets if appropriate landmarks (such as multiple host-implant bridges) can be identified for 3D registration. Further development of imaging techniques should aim to enable dynamic longitudinal studies and flow quantification of remodeling vasculature.

Limited literature examines the impacts of in vitro perfusion and in vivo vascularization on hiPSC-CM structural and functional maturation [8,22,78], despite developmental evidence suggesting cardiac maturation is intimately coupled with coronary growth [79,80]. To begin to address the hypothesis that co-revascularization with remuscularization by vEHM implantation improves engrafted hiPSC-CM development and maturation, we pursued bulk RNAseq analysis on explanted EHMs after 2 weeks in vivo to explore early genotype shifts. Despite experimental challenges that resulted in poor human gene alignment and thus a subpar sample size for proper DEG analysis, our preliminary analysis with equal variance assumed showed upregulation of genes related to cardiac development in vEHMs relative to controls. Although inconclusive given the sample size and analysis methodology, our preliminary results show promise for pursuing RNAseq to elucidate mechanisms of vascular-driven growth and maturation. While other groups have shown RNAseq analyses of cardiomyocytes in 2D and 3D in vitro tissues for cardiac applications [13,29,81], as well as whole-heart bulk and single-cell RNA sequencing [82,83,84], only one group has very recently published bulk RNA sequencing of intramyocardially injected hiPSC-CMs in a rodent model of I/R MI [85]. To the best of our knowledge, this is the first report on bulk RNA sequencing of explanted cardiac tissues from a multi-species preclinical model. Future work aims to optimize isolation procedures to ensure high-quality data for bulk or single cell/nuclei RNAseq and pursue multi-species alignment deconvolution [86] for advancing assessment of host-implant heterocellular vascular interactions and their contributions to cardiac regeneration. Pursuing these types of methodologies to understand mechanistic insights at multiple time points will enable us to answer emerging questions, such as how hiPSC-CM development and maturation are facilitated by inosculation at earlier timepoints and what the contributions of a co-revascularization strategy with remuscularization are for long-term in vivo maturation and durable heart regeneration.

## 5. Conclusions

In summary, we developed a flexible in vitro system for fabrication and dynamic perfusion culture of vEHMs and demonstrated successful inosculation and hierarchical vascular bed development in a remuscularization approach to heart regeneration using a rat model of ischemia/reperfusion MI. Implantation of vEHMs revealed that endothelialized channels contributed to increased vessel diameter and branching within implants. In vitro perfusion of vEHMs prior to implant provided the greatest improvements in hiPSC-CM engraftment, organization of the vasculature for efficient hierarchical perfusion by increasing diameters and reducing tortuosity, recruitment of SMA+ vascular support cells, and potentially also hiPSC-CM development. This study increases our understanding of the design criteria for fabricating patterned vEHMs and their direct benefits on engineered tissue survival in vivo in a co-vascularization-remuscularization strategy for therapeutic regeneration of the heart after MI.

## 6. Patents

This work resulted in the submission of a provisional patent with the US Patent and Trademark Office (63/369,338, filed 7/25/23).

## Data Availability

Data will be made available upon request.

## Supplementary Materials

The following supporting information can be downloaded at: https://www.mdpi.com/article/10.3390/cells12131698/s1, Table S1: Antibodies used in immunohistochemical staining; Figure 1: Cellular viability and morphology; Figure S2: EHM compaction and contractile kinetics; Figure S3: Wet-spun collagen microfiber bundles; Figure S4: Echocardiographic metrics of heart function; Figure S5: Characterization of human cells in implants; Figure S6: Implant vascularization and human vascular morphology; Figure S7: Perfusion quantification by OCT and microCT; Figure S8: Exploratory bulk RNA sequencing of explanted EHMs. Video S1: hiPSC-CMs at day 30 after expansion and lactate selection; Video S2: EHM at day 5 prior to implantation; Video S3: Vascular reconstruction of a microCT-scanned heart with a representative EHM implant; Video S4: Vascular reconstruction of a microCT-scanned heart with a representative NP vEHM implant; Video S5: Vascular reconstruction of a microCT-scanned heart with a representative P vEHM implant.
