# Supplementary material for: Patterned Arteriole-Scale Vessels Enhance Engraftment, Perfusion, and Vessel Branching Hierarchy of Engineered Human Myocardium for Heart Regeneration
Source: Cells. 2023 Jun 23;12(13):1698. doi: 10.3390/cells12131698 (PMC10340601; doi:10.3390/cells12131698)
Supplement: Supplementary file 1 [file cells-12-01698-s001.zip › cells-2421724-supplementary.pdf]

Antibody/Fluorophore	Dilution	Catalog No.; Vendor
Mouse monoclonal anti-α sarcomeric actinin	1:500	A7811-.2ML; MilliporeSigma
Mouse monoclonal anti-cardiac troponin T	1:100	MA5-12960 UL; Invitrogen
Rabbit monoclonal anti-smooth muscle actin	1:150	ab150301; Abcam
Mouse monoclonal anti-smooth muscle actin	1:150	PIMA511547; Invitrogen
Mouse monoclonal anti-human CD31	1:100	M082301-2; Agilent
Rabbit polyclonal anti-Myosin light chain 2v	1:100	10906-1-AP; ProteinTech
Mouse monoclonal anti-Myosin light chain 2a	1:100	311 011; Synaptic Systems
Rabbit polyclonal anti-cardiac troponin I	1:150	ab47003; Abcam
Rabbit polyclonal anti-Connexin-43	1:200	C6219-100UL; MilliporeSigma
Rabbit monoclonal anti-human Ku80	1:150	2180S; Cell Signaling Technologies

Bisbenzimide H 33342 trihydrochloride (Hoechst)	1.5 μg/mL	B2261-100MG; MilliporeSigma
Goat anti-mouse/rabbit Alexa Fluor 488	1:300	A-1100; A-11008; Invitrogen
Goat anti-rabbit/mouse Alexa Fluor 594	1:300	A11012; A-11005; Invitrogen
Griffonia simplicifolia isolectin-B4 Dylight 594-labeled	1:200	DL-1207-.5; Vector Labs
Mouse anti-human Ki67 FITC-labeled	1:50	612472; BD Biosciences

Table S1. Antibodies used in immunohistochemical staining.

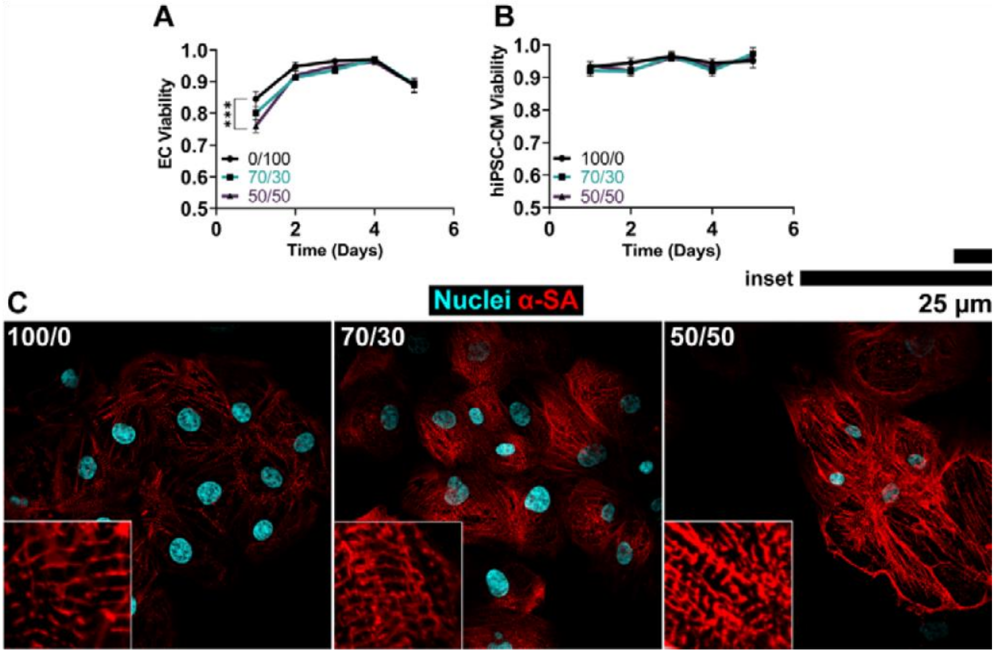

Figure S1: Cellular viability and morphology. **(A)** EC and **(B)** hiPSC-CM viability in response to culture in mixed media. n = 6-9 per group. **(C)** Histological staining for α-sarcomeric actinin in hiPSC-CMs. α-SA: α-sarcomeric

actinin. Mixed media formulations are indicated as percent B27/percent EGM-2 (e.g., 70/30: 70% B27 and 30% EGM-2). *** $p < 0.001$.

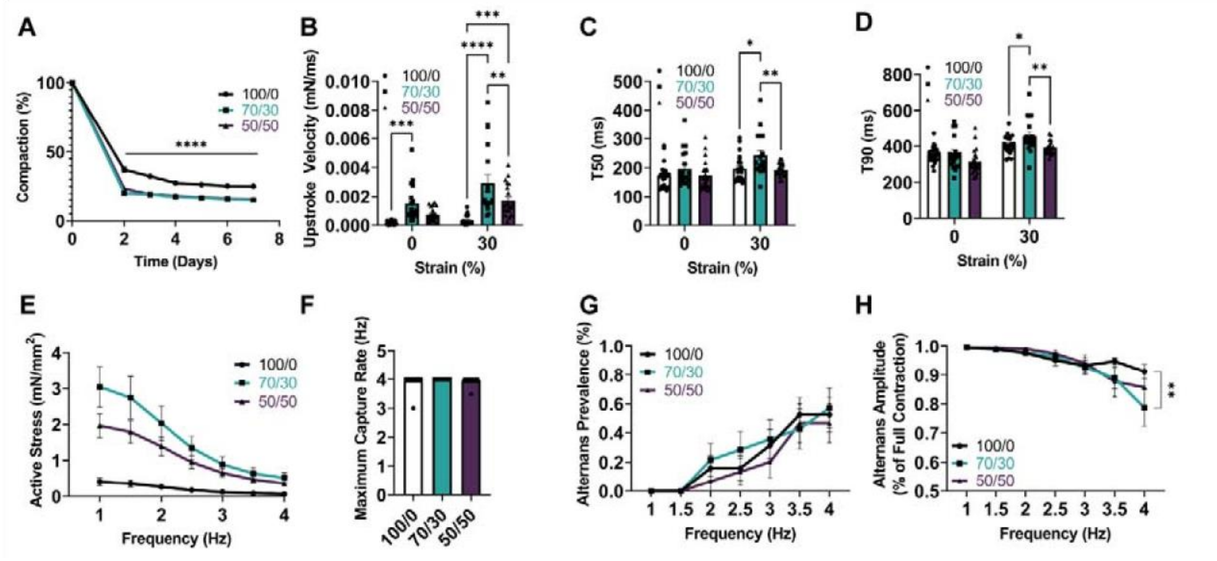

Figure S2: EHM compaction and contractile kinetics. **(A)** EHM compaction over one week of mixed media culture. EHM contractile kinetics of **(B)** upstroke velocity, time to **(C)** 50% and **(D)** 90% relaxation (T50 and T90), **(E)** force frequency response, and **(F)** maximum capture rate. $n = 18\text{-}21$ per group. **(G)** Prevalence of contractile alternans and **(H)** relative amplitude of partial-force contractions observed during force-frequency response testing. $n = 14\text{-}19$ per group. Mixed media formulations are indicated as percent B27/percent EGM-2 (e.g., 70/30: 70% B27 and 30% EGM-2). * $p < 0.05$; ** $p < 0.01$; *** $p < 0.001$; **** $p < 0.0001$.

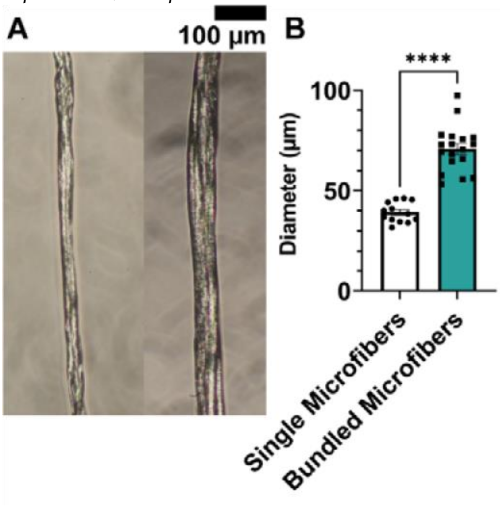

Figure S3: Wet spun collagen microfiber bundles. **(A)** Wet spun collagen microfibers are wrapped around themselves four times to create bundled fibers. **(B)** Collagen microfiber diameter compared to individual microfibers. $n = 16\text{-}18$ per group. **** $p < 0.0001$.

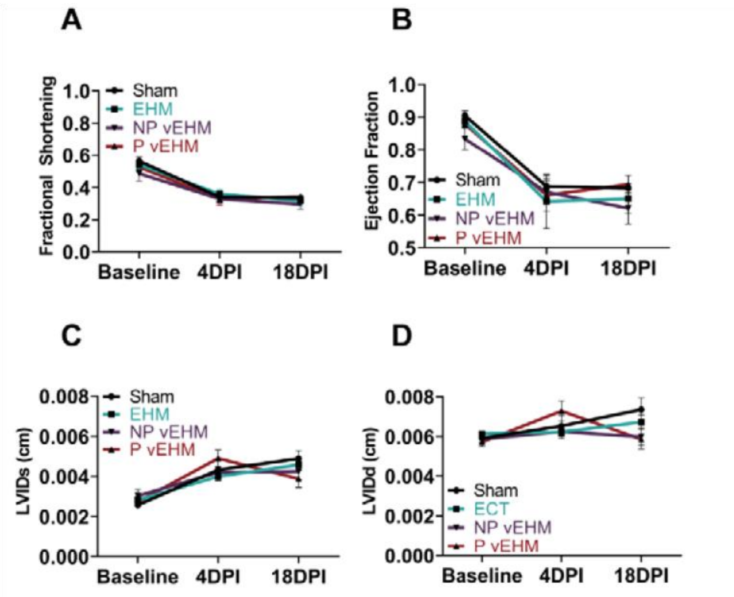

Figure S4: Echocardiographic metrics of heart function. **(A)** Measurements of fractional shortening, **(B)** ejection fraction, and left ventricular inner diameter in **(C)** systole (LVIDs) and **(D)** diastole (LVIDd). *n* = 4-6 per group.

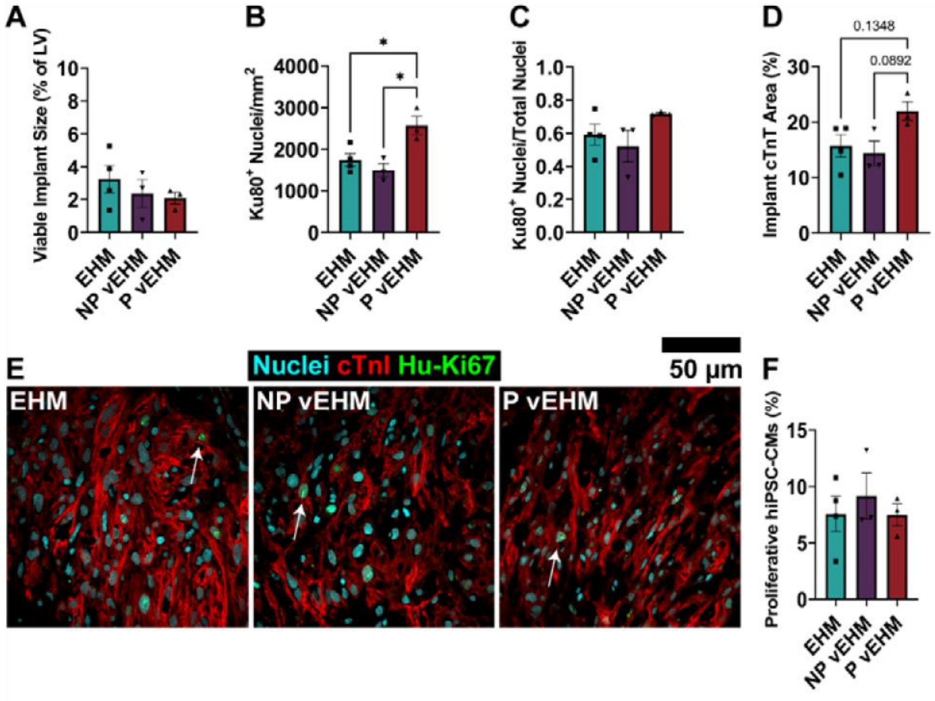

Figure S5: Characterization of human cells in implants. **(A)** Viable implant size relative to LV size. **(B)** Density of human cells in implants by Hu-Ku80+ staining. **(C)** Ratio of Hu-Ku80+ to total nuclei. **(D)** Measurement of cTnT

area in implants. **(E, F)** Proliferation assessment of implanted hiPSC-CMs by staining for Hu- Ki67 (white arrows) and cTnI. n = 3-4 per group. cTnT: cardiac troponin T; Hu-Ki67: Human-specific Ki67. * $p < 0.05$.

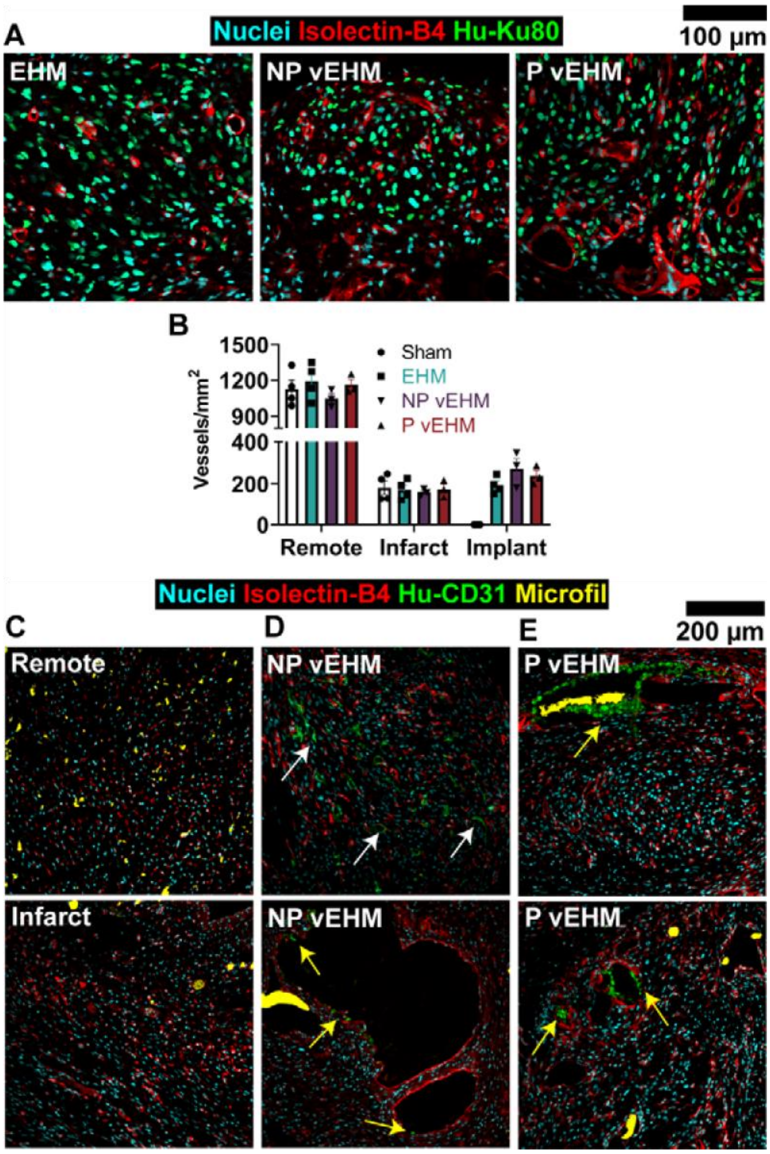

Figure S6: Implant vascularization and human vascular morphology. **(A)** Representative images of vessel morphology in implants. **(B)** Quantification of number of vessels across remote, infarct, and implants regions. n = 3-4 per group. **(C)** Representative vascular stains of the remote and infarct regions of sham hearts. **(D)** Human ECs in NP vEHMs appear as isolated capillary sprouts (white arrows) or as part of larger, host-lined vessels (yellow arrows). **(E)** Human ECs in P vEHMs are only found in chimeric vessels (yellow arrows). Hu-Ku80: Human-specific Ku80; HuCD31: Human-specific CD31.

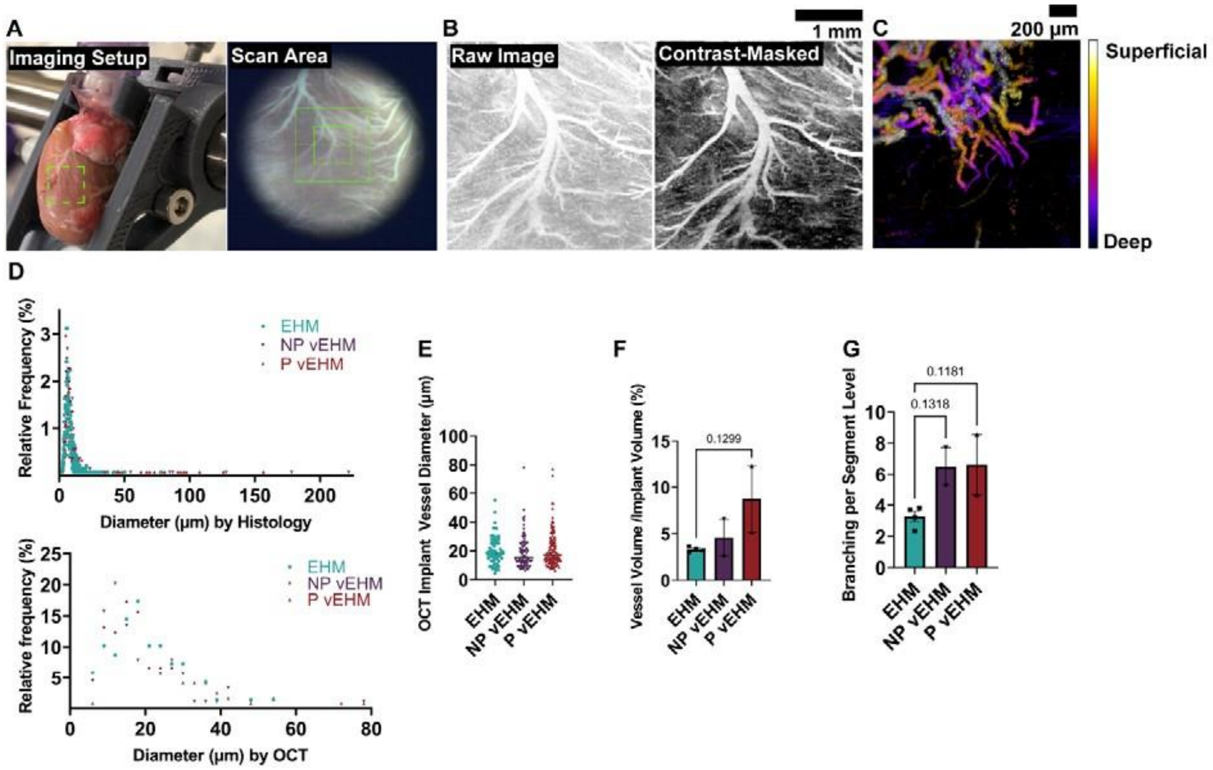

Figure S7: Perfusion quantification by OCT and microCT. **(A)** OCT imaging setup during intralipid perfusion and example scan area (green dotted square) with 5x objective. **(B)** Corresponding preview image after acquisition and final image after contrast-mask averaging of multiple volumes. **(C)** False-colored image of implant vasculature demonstrating vessel depth. **(D)** Comparison of vessel diameters as measured by histological analysis and **(E)** OCT. MicroCT analysis of **(F)** intra-implant vascular volume and **(G)** branching per segment level. n = 2-3 per group. OCT: Optical Coherence Tomography.

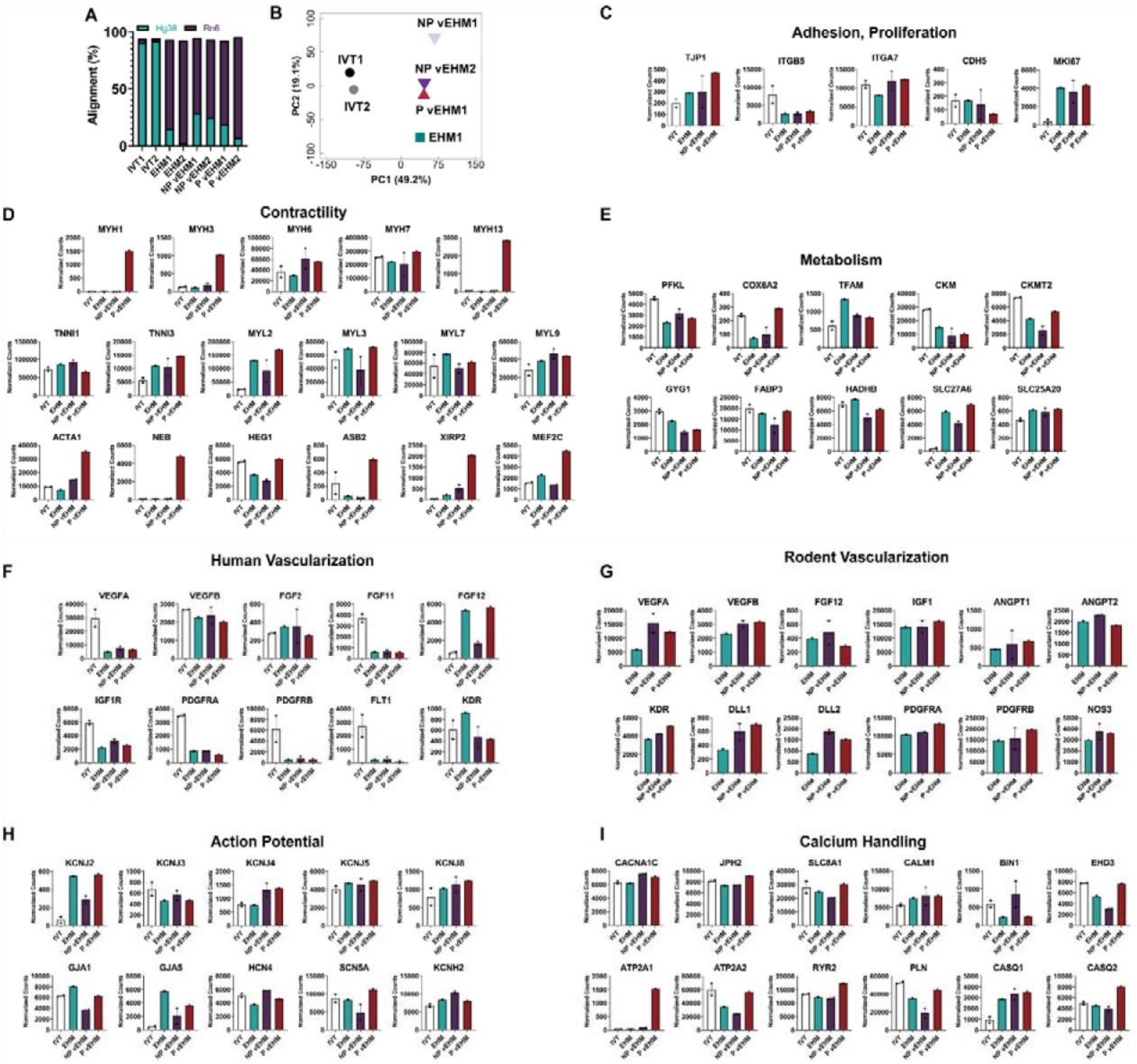

Figure S8: Exploratory bulk RNA sequencing of explanted EHMs. **(A)** Alignment of sequenced samples to hg38 (human) and rn6 (rat) genomes. **(B)** Principal component analysis of human-aligned samples. **(C-I)** Comparison of gene regulation across categories of cardiomyocyte and vascular development. *n* = 1-2 per group.

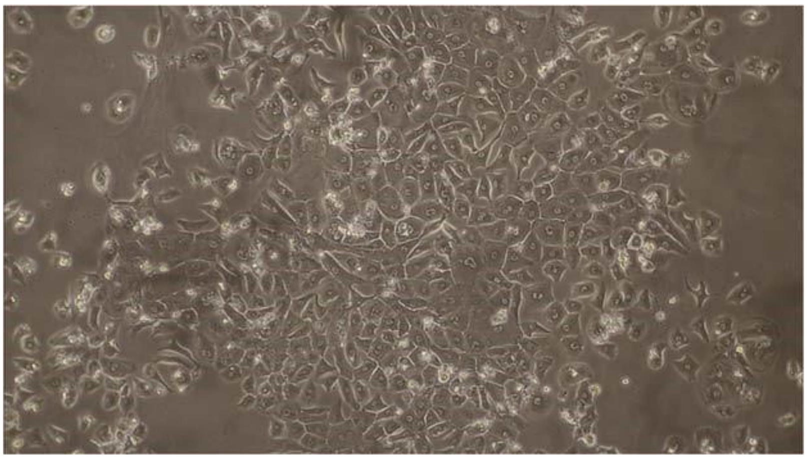

Suppl. Video S1: hiPSC-CMs at day 30 after expansion and lactate selection.

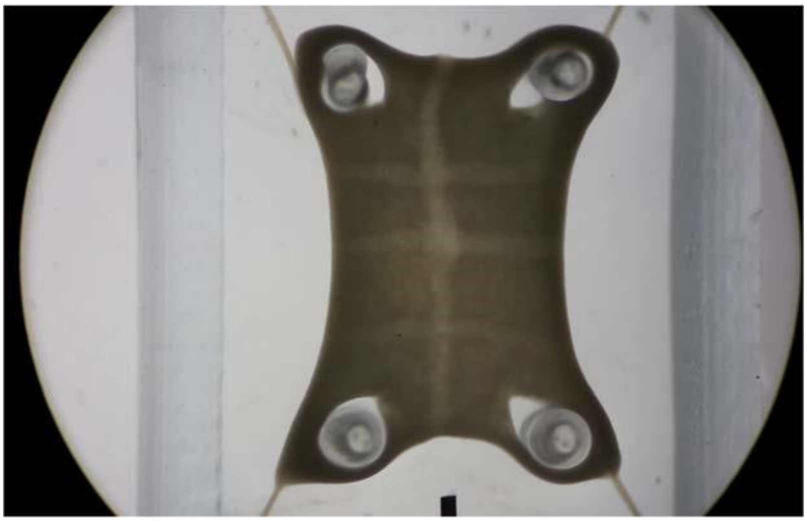

Suppl. Video S2: EHM at day 5 prior to implantation.

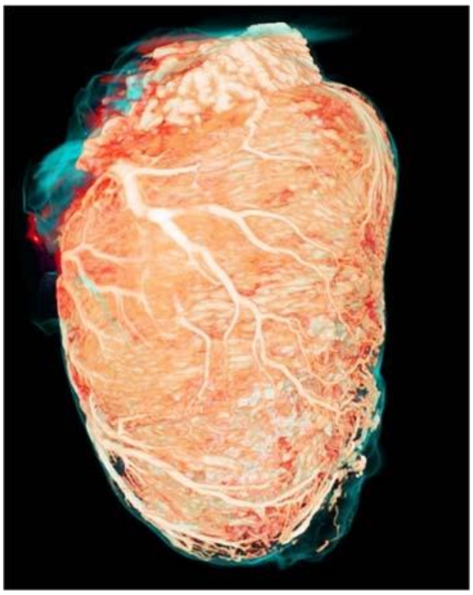

Suppl. Video S3: Vascular reconstruction of microCT-scanned heart with representative EHM implant.

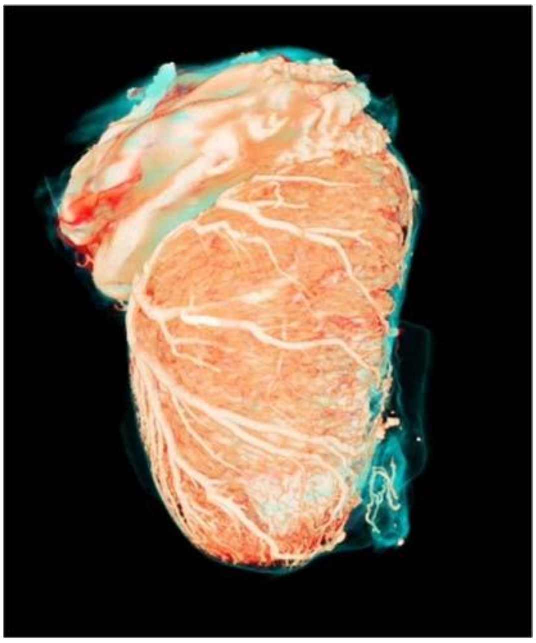

Suppl. Video S4: Vascular reconstruction of microCT-scanned heart with representative NP vEHM implant.

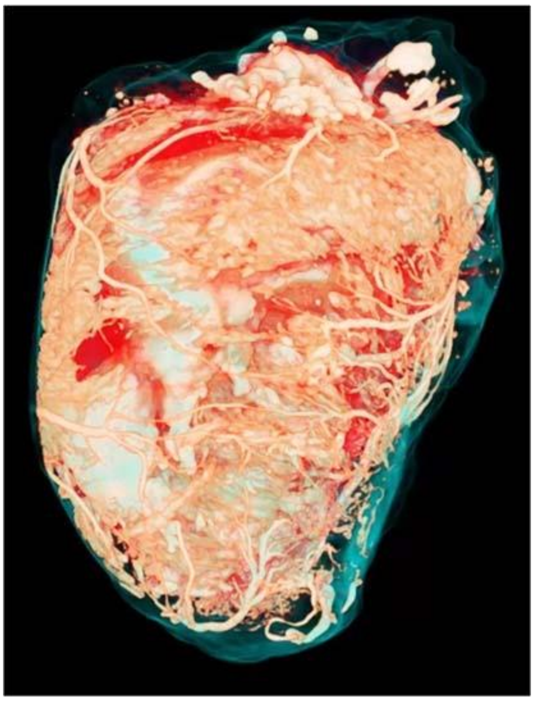

Suppl. Video S5: Vascular reconstruction of microCT-scanned heart with representative P vEHM implant.
