# Supplementary figures and images for: Patterned Arteriole-Scale Vessels Enhance Engraftment, Perfusion, and Vessel Branching Hierarchy of Engineered Human Myocardium for Heart Regeneration
Source: Cells. 2023 Jun 23;12(13):1698. doi: 10.3390/cells12131698 (PMC10340601; doi:10.3390/cells12131698)

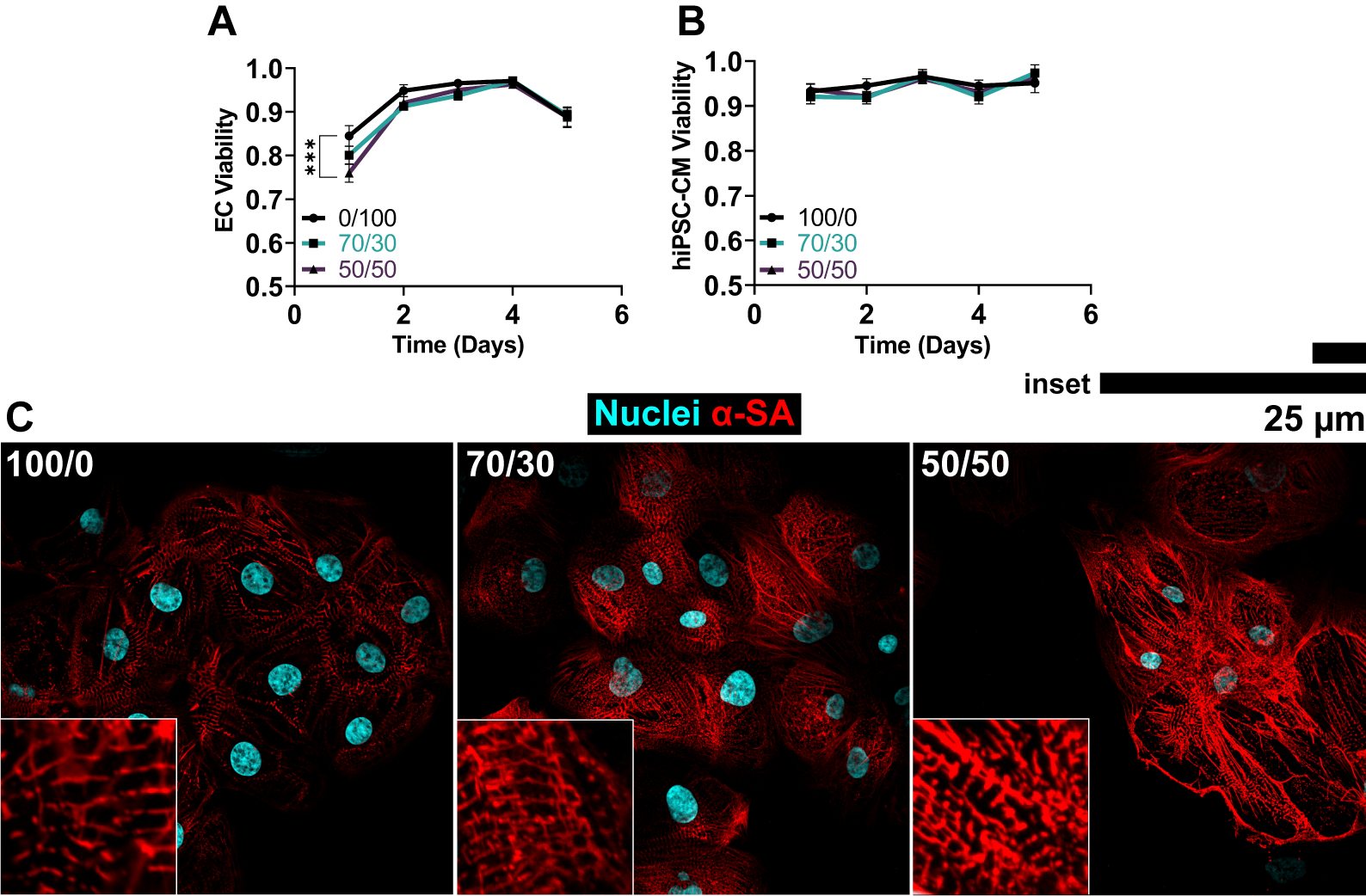

Supplement: Supplementary file 1 [file cells-12-01698-s001.zip › SupplFig1.tif]

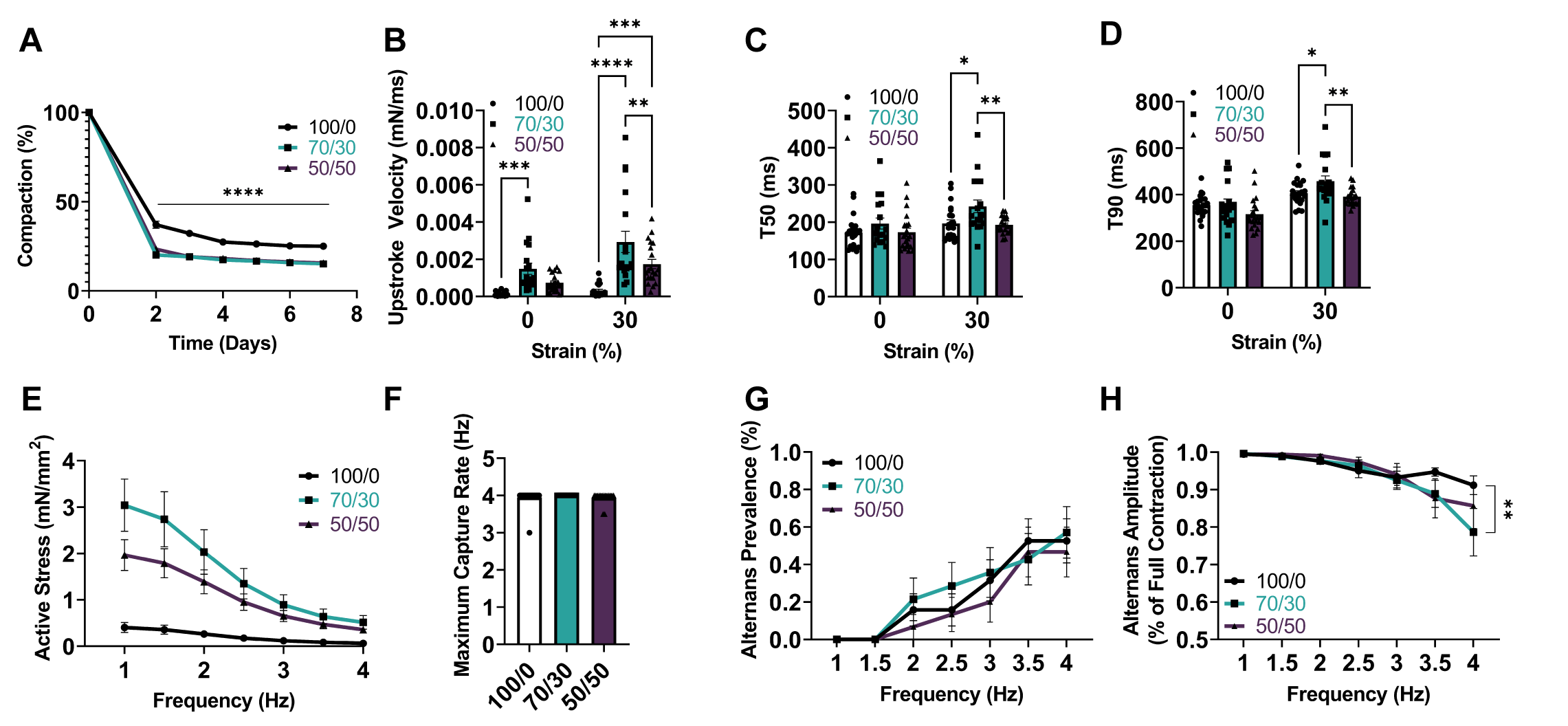

Supplement: Supplementary file 1 [file cells-12-01698-s001.zip › SupplFig2v2.tif]

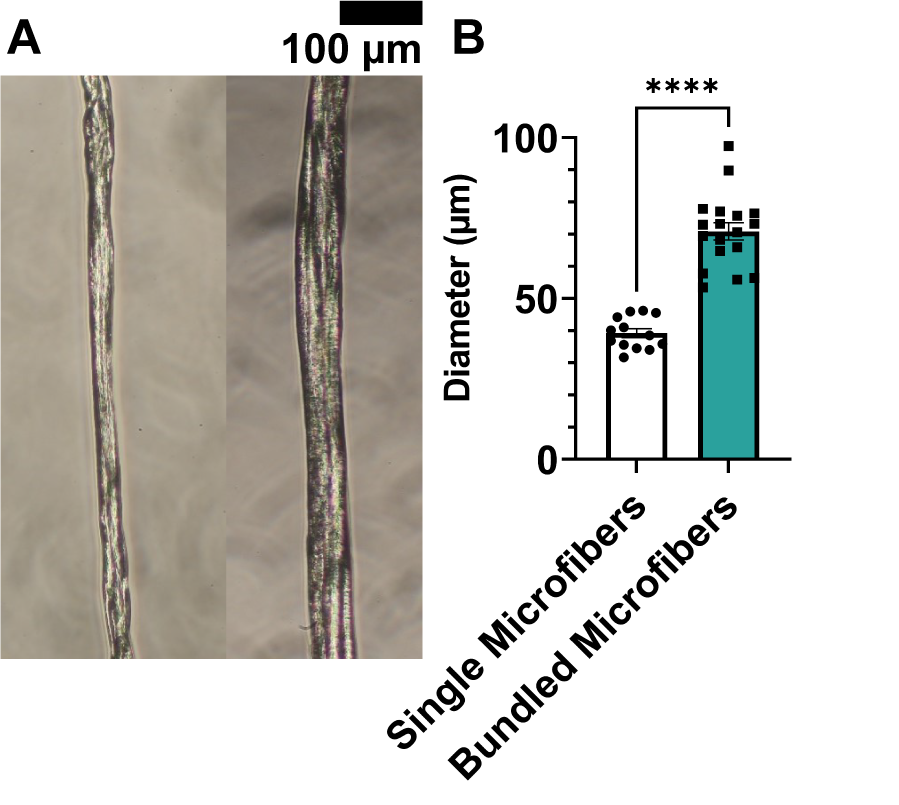

Supplement: Supplementary file 1 [file cells-12-01698-s001.zip › SupplFig3v2.tif]

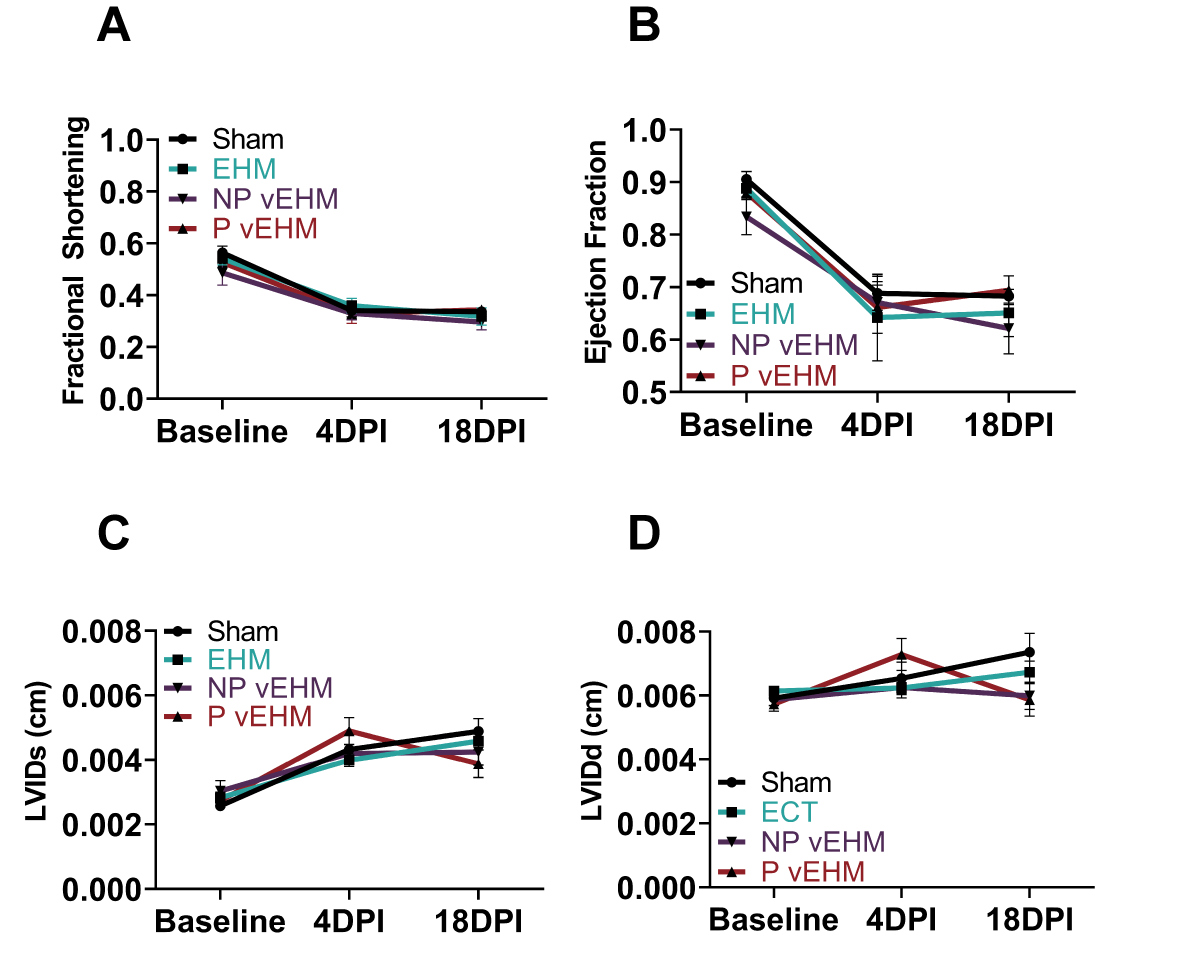

Supplement: Supplementary file 1 [file cells-12-01698-s001.zip › SupplFig4.tif]

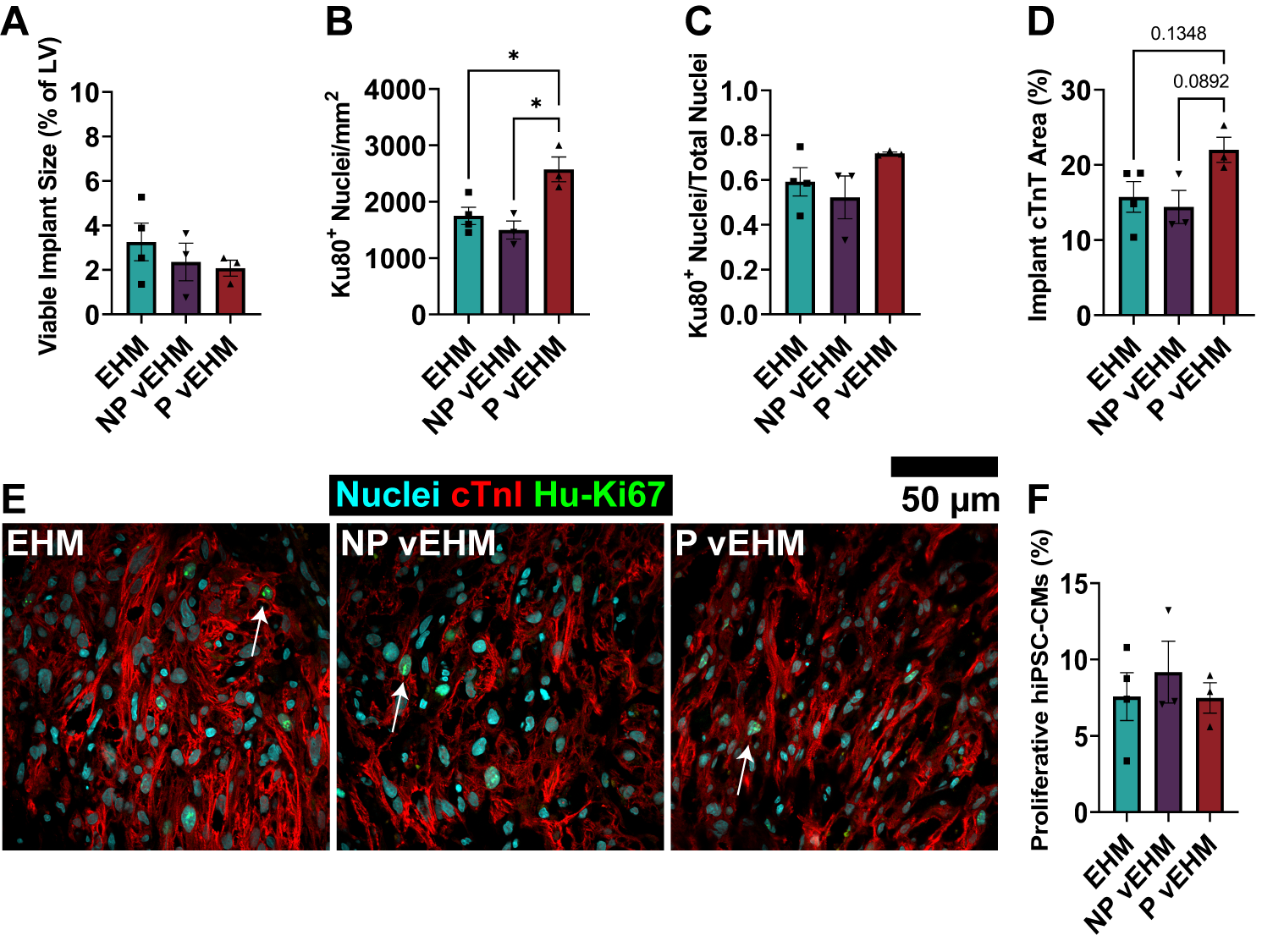

Supplement: Supplementary file 1 [file cells-12-01698-s001.zip › SupplFig5v4.tif]

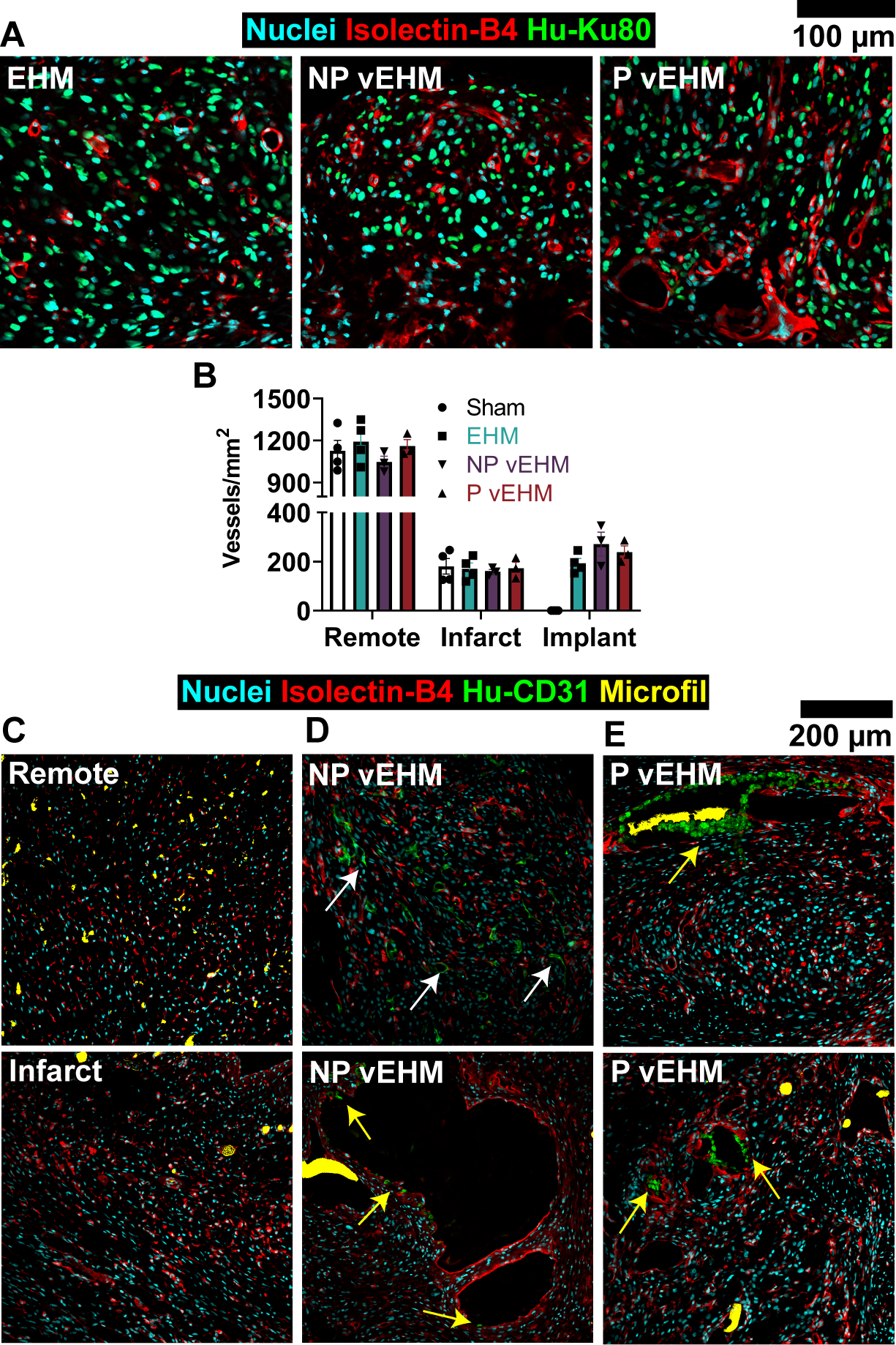

Supplement: Supplementary file 1 [file cells-12-01698-s001.zip › SupplFig6V2.tif]

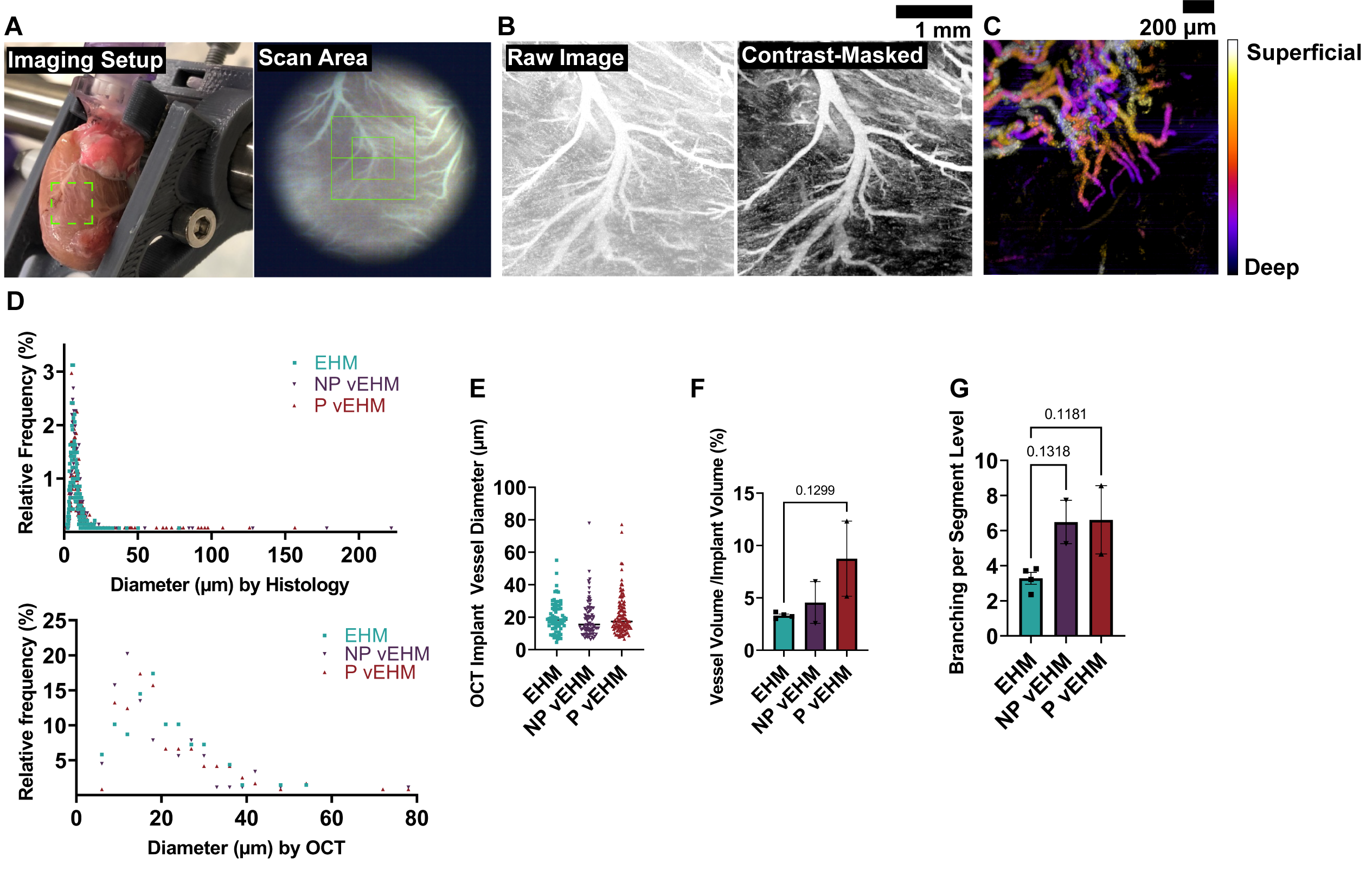

Supplement: Supplementary file 1 [file cells-12-01698-s001.zip › SupplFig7v2.tif]

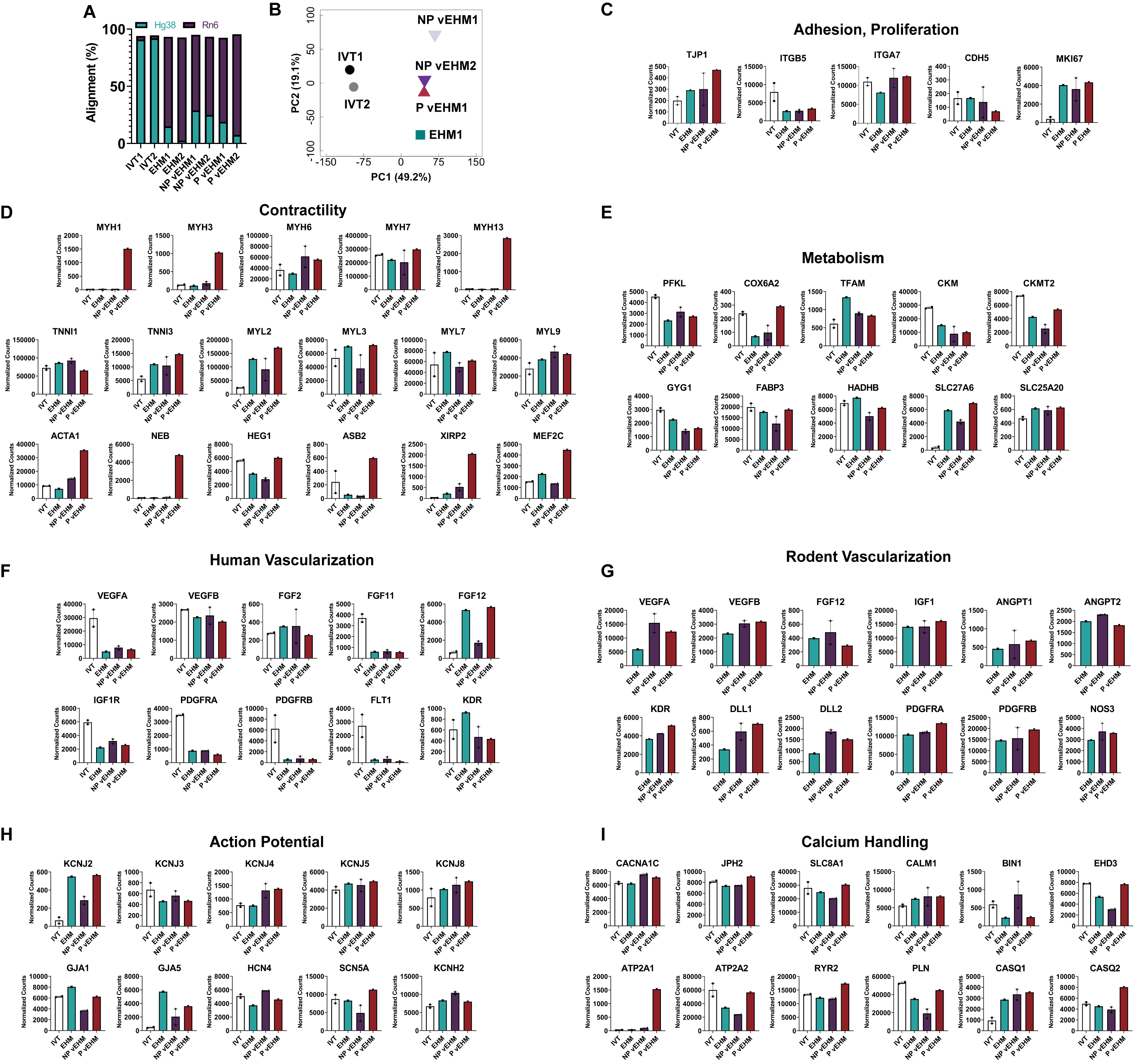

Supplement: Supplementary file 1 [file cells-12-01698-s001.zip › SupplFig8.tif]
